# Gene expression patterns in four brain areas associate with quantitative measure of estrous behavior in dairy cows

**DOI:** 10.1186/1471-2164-12-200

**Published:** 2011-04-19

**Authors:** Arun Kommadath, Henri Woelders, Bonne Beerda, Herman A Mulder, Agnes AC de Wit, Roel F Veerkamp, Marinus FW te Pas, Mari A Smits

**Affiliations:** 1 Animal Breeding and Genomics Centre, Wageningen UR Livestock Research, P.O. Box 65, 8200 AB Lelystad, The Netherlands; 2 Adaptation Physiology Group, Department of Animal Sciences, Wageningen University, 6709 PG Wageningen, The Netherlands

## Abstract

**Background:**

The decline noticed in several fertility traits of dairy cattle over the past few decades is of major concern. Understanding of the genomic factors underlying fertility, which could have potential applications to improve fertility, is very limited. Here, we aimed to identify and study those genes that associated with a key fertility trait namely estrous behavior, among genes expressed in four bovine brain areas (hippocampus, amygdala, dorsal hypothalamus and ventral hypothalamus), either at the start of estrous cycle, or at mid cycle, or regardless of the phase of cycle.

**Results:**

An average heat score was calculated for each of 28 primiparous cows in which estrous behavior was recorded for at least two consecutive estrous cycles starting from 30 days post-partum. Gene expression was then measured in brain tissue samples collected from these cows, 14 of which were sacrificed at the start of estrus and 14 around mid cycle. For each brain area, gene expression was modeled as a function of the orthogonally transformed average heat score values using a Bayesian hierarchical mixed model. Genes whose expression patterns showed significant linear or quadratic relationships with heat scores were identified. These included genes expected to be related to estrous behavior as they influence states like socio-sexual behavior, anxiety, stress and feeding motivation (*OXT, AVP, POMC, MCHR1*), but also genes whose association with estrous behavior is novel and warrants further investigation.

**Conclusions:**

Several genes were identified whose expression levels in the bovine brain associated with the level of expression of estrous behavior. The genes *OXT *and *AVP *play major roles in regulating estrous behavior in dairy cows. Genes related to neurotransmission and neuronal plasticity are also involved in estrous regulation, with several genes and processes expressed in mid-cycle probably contributing to proper expression of estrous behavior in the next estrus. Studying these genes and the processes they control improves our understanding of the genomic regulation of estrous behavior expression.

## Background

Maintaining good fertility and thereby optimum reproductive performance in dairy cows is of great economic importance for the dairy industry. Knowledge on factors influencing fertility is already being applied to improve or regulate fertility. For example, the importance of limiting negative energy balance in early lactation cows for proper reproductive performance is well recognized [1,2]. Insight into the hormonal regulation of estrous cycle has found practical application to artificially regulate the cycle in farm animals and to manage or treat fertility related problems. However, current understanding of genomic factors underlying fertility is limited and this obstructs the development of novel genomic tools and managemental strategies for improving and optimizing reproductive performance, such as biomarkers to monitor the fertility status of cows. Studying the genomic factors underlying fertility may help to optimize nutritional or management systems that improve reproductive performance [3] and also to explain the genetic basis for the decline in several fertility traits of high producing dairy cows. Currently it is known that this decline may be partly attributed to physiological adaptations by the cow to high milk production [4].

Among the fertility traits, the expression of estrous behavior (heat), a key fertility trait that marks the fertile period in cows, has decreased both in duration and intensity over generations of cows selected for high milk yield [5]. Short heat periods and the absence of clear behavioral signs of heat cause farmers to fail to detect heat or to misjudge the optimum time of insemination of their cows, resulting in financial losses due to prolonged interval from calving to first insemination, reduced conception rates and increased calving intervals.

In an effort to understand the genomic regulation of estrous behavior expression in dairy cows, a microarray experiment was set up to study gene expression levels in 4 different brain areas and the anterior pituitary of cows sacrificed at either the start of estrus (day0 of estrous cycle) or at mid-cycle (day12). Differential gene expression analysis between day0 and day12 cows for each of these tissues revealed a limited number of significant genes in the anterior pituitary alone and none in the brain areas (detailed results not reported here). When the trait of interest is quantitative, as in this case (estrous behavior quantified as heat score), the grouping of individuals into qualitative classes dilutes the available information. Therefore, the association between gene expression and phenotypic trait may be better analyzed using the individual quantitative measurements. Using this approach in an earlier study, we identified a set of a few hundred probes out of approximately 24,000 probes on a bovine microarray corresponding to genes whose level of expression in the anterior pituitary of experimental cows associated with the degree to which the individual cows expressed estrous behavior [6]. Among these probes were genes encoding hormones like FSH and prolactin, whose roles in estrous regulation are well-known. Further, biological processes relevant to estrous behavior were over-represented in this set of genes. These results give confidence in the association analysis methodology followed, though experimental validation is needed to determine to what extent the associated genes regulate estrous behavior. In addition to the anterior pituitary, it is likely that a number of areas of the cow brain also have genes whose levels of expression associate with the degree to which cows express estrous behavior. Studies in rodents have revealed that estrogen dependent female reproductive behavior happens via well orchestrated genomic responses in the forebrain with the hypothalamus playing a major role [7,8]. Areas in the limbic region of the forebrain like the amygdala and hippocampus were found to have functions related to sexual behavior and associated emotional responses [9,10]. As yet, there have been no studies in cows linking gene expression in the brain to estrous behavior. Identifying and studying genes whose level of expression in different brain areas of cows associate with the degree to which these cows express estrous behavior will help improve our understanding of genomic factors underlying fertility.

The objective here is to identify and study those genes that associated with estrous behavior, among genes expressed in four bovine brain areas (hippocampus, amygdala, dorsal hypothalamus and ventral hypothalamus), either at the start of estrous cycle, or at mid cycle, or regardless of the phase of cycle.

## Results

The degree of estrous behavior expression was quantified as a cow's average heat score using heat scores recorded from at least two consecutive cycles (Table 1). The data from one of the day0 cows was excluded from further analysis because of its high outlier value of 1750 for average heat score, which we attributed to that cow's several consecutive attempts to mount other cows during one observation period. The average heat scores for the remaining 13 day0 cows ranged from 0 to 405, with a mean value of 178.4 (SD 125.7), and the average heat scores for the 14 day12 cows ranged from 2 to 505, with a mean value of 244.7 (SD 175.4). The average heat scores were used with the corresponding gene expression data to run the three analyses per brain area as summarized in Table 2.

**Table 1:** Average heat scores of the experimental cows sacrificed at day0 or day12 of estrous cycle

day0 Cow Nr	Heat score	day12 Cow Nr	Heat score
d0_5006	0	d12_1528	2
d0_9284	37	d12_9303	4
d0_3739	43	d12_8860	5
d0_8855	53	d12_1773	75
d0_1194	137	d12_8873	157
d0_1821	175	d12_7724	198
d0_8870	191	d12_1520	257
d0_5507	200	d12_7942	275
d0_1786	206	d12_1607	318
d0_3472	246	d12_1822	368
d0_6487	248	d12_1638	383
d0_3747	378	d12_6956	404
d0_7008	405	d12_8857	475
d0_5125 *	1750	d12_2540	505

* Data from this cow was not used in the analysis due to outlier heat score value

**Table 2:** Description of the three analyses and their objectives

Analysis	Data	Objective
day0	Gene expression data from the tested brain area of Day0 cows and their average heat scores	To identify genes of which the expression in the tested brain area at the start of estrus was associated with estrous behavior
day12	Gene expression data from the tested brain area of Day12 cows and their average heat scores	To identify genes of which the expression in the tested brain area around mid cycle (diestrus) was associated with estrous behavior
day0 + day12	Gene expression data from the tested brain area of Day0 and Day12 cows and their average heat scores	To identify genes of which the expression in the tested brain area was associated with estrous behavior regardless of the phase of estrous cycle

**Figure 1:**
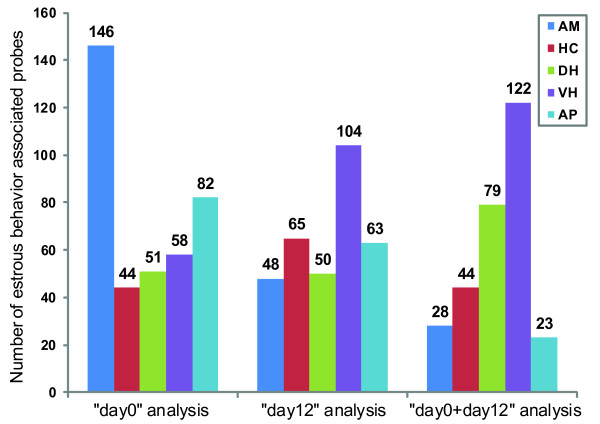
**Number of estrous behavior associated probes found in 4 brain areas and anterior pituitary**. AM - Amygdala; HC - Hippocampus; DH - Dorsal Hypothalamus; VH - Ventral Hypothalamus; AP - Anterior Pituitary.

**Figure 2:**
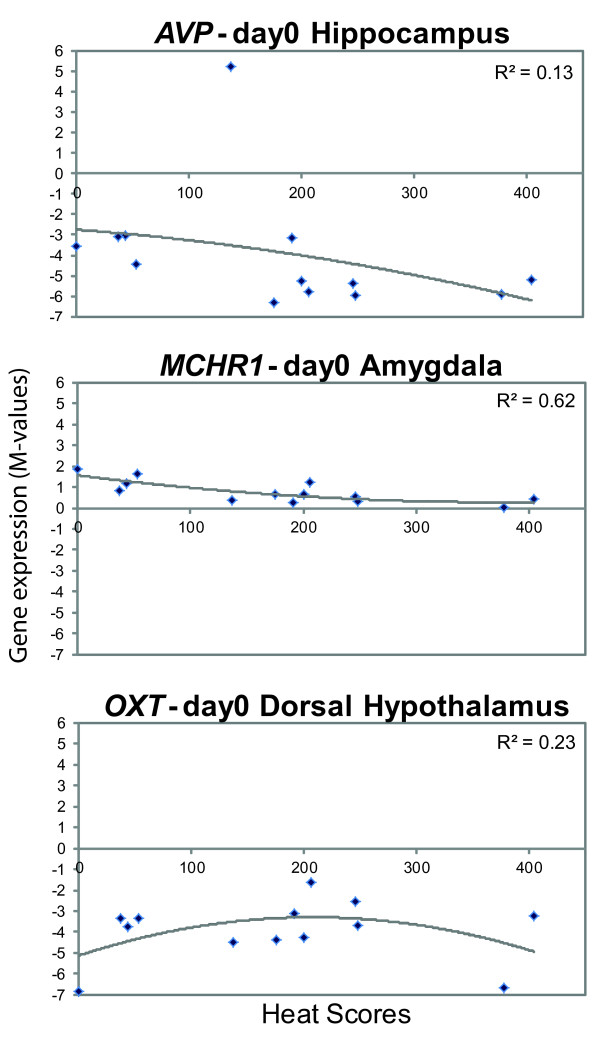
Association patterns of gene expression profile with heat scores for three estrous behavior associated genes at day0.

Additional file 1 lists the associated genes found in each analysis and describes the pattern of association between gene expression and heat score for each of the genes in the list. The patterns noticed were: linear (positive or negative slopes) or quadratic (positive/convex shaped or negative/concave shaped curves). The total number of heat score associated probes found common to all 5 Gibbs sampling chains per analysis in each brain area is provided in Figure 1. Additional file 2 depicts Venn diagrams that show the number of overlapping probes between the different brain areas per analysis. The overlap was highest between the DH and VH and then between the AM and HC. The figure 1 and the additional file 2 includes the results of the re-analysis on the AP as well, for which the number of associated probes found for the three analyses (82, 63 and 23 for day0, day12 and day0+day12 respectively) were now considerably lower than the numbers (177, 142 and 118 for day0, day12 and day0+day12 respectively) from the association analysis with third order polynomials and one Gibbs sampling chain per analysis as done earlier [6]. For the different brain areas, the percentage of associated probes found common to all chains per analysis varied from 50-80%. For day0, a relatively high number of heat score associated probes were detected for AM and AP (146 and 82 respectively) whereas for day12 this was true for VH (104). For DH, the numbers were approximately equal at day0 and day12 (51 and 50 respectively). Figure 2 provides the association patterns for three genes whose expression values were found to be associated with heat score at day0. For *AVP *and *MCHR1*, a linear trend was observed in HC and AM respectively whereas for *OXT*, a quadratic trend was observed in DH.

Of the total 640 unique probes found associated with heat score in all analyses and brain areas, 372 had gene annotations. Sets of genes associated with functional categories which we group as: "transcription and regulation of gene expression", "detection of and responses to stimuli" and "signaling pathways" each made up about 15% of the genes that were identified. In the category "detection of and responses to stimuli" we included the genes involved in neurotransmission through encoding for neurotransmitters/hormones (*OXT, POMC, AVP, CCK, CGA*) or neurotransmitter receptors (*GABRA6, HTR2A, MCHR1, CHRM1, CHRM3, DRD2, CHRNA5*) and metabolizing enzymes (*PTGDS, PTGIS, PTGR1, ACHE, SULT4A1*). We could also identify 5 more sets of genes, each consisting of between 5 to 10% of the total genes. These included genes associated with: "transport and localization" (positioning of a substance or cellular entity, like sorting nexin family member genes), "transporter activity" (e.g. calcium channels and Na+/K+ transport), "metabolism" (e.g. glucose and amino acid metabolism or steroidogenesis), "cell cycle" (here including processes linked to DNA folding and repair) and "multi-organism processes". In the last category we included immune system related genes like *CTLA4, IL1RL1, MARCO, FCRLA *and *IL33*.

A detailed list of all significant enriched GO and KEGG pathway terms (p < 0.10) found in the different brain areas and analyses is provided in Additional file 3. For illustration, a summary of only those GO biological processes that cleared a FDR cutoff of 20% is provided in Table 3. Several relevant processes related to behavior, neurotransmission and signaling, and ion regulation were found especially in day0. The presence of enriched processes related to behavior like 'grooming behavior', 'regulation of female receptivity' and 'female mating behavior' within the associated genes found in HC indicates the key role of the genes *AVP *and *OXT *that contribute to these processes. Terms related to neurotransmission and signaling and the associated ion regulation terms too have biological implications related to estrous behavior as evident from the discussion below on some of the genes that contribute to these processes.

**Table 3:** Significantly enriched Gene Ontology Biological Process terms in the estrous behavior associated gene lists*

Gene Ontology Biological Process term	day0	day12	day0 + day12
**Behavior related terms**			
regulation of female receptivity; reproductive behavior in a multicellular organism; female mating behavior; maternal aggressive behavior; maternal behavior; parental behavior	HC	DH	HC
grooming behavior			HC
locomotory behavior	AP^+^		
behavior	AP^+^		

**Neurotransmission and signaling related terms**			
neurological system process	AP^+^		
regulation of neurotransmitter levels	AP		
transmission of nerve impulse	AP^+^		
regulation of synaptic transmission	DH		
cell surface receptor linked signal transduction		DH^+^	
cell-cell signaling	HC^+^		
second-messenger-mediated signaling			VH^+^
response to cAMP	DH		
Wnt receptor signaling pathway	DH		
phosphoinositide-mediated signaling			VH

**Ion regulation related terms**			
ion transport	AP^+^		
elevation of cytosolic calcium ion concentration			DH
negative regulation of ion transmembrane transporter activity	AM		
cytosolic calcium ion homeostasis	AM		

* Only terms with p < 0.10 and fdr <0.20 are included. Terms typical of other organs (these terms appeared here due to genes with different functions in different organs) have been removed from this summary table. Where the term is supported by 5 or more genes in the gene list, the corresponding brain area is followed by a ^**+ **^sign whereas in cases where the term is supported by only 2-4 genes, the sign is absent.

## Discussion

Variation in the behavioral trait to express estrous behavior was found to be associated with variation in the expression of genes in the cow's brain areas: DH, VH, HC and AM and also the AP, an endocrine gland pivotal in synchronizing estrous behavior with hormonal and ovarian events preceding ovulation. The choice of these tissues for this study was based on their reported involvement in regulating female sexual behavior. Although genes differ in their influence on specific traits, it is tempting to consider the AM to be of relatively higher importance for regulating estrous behavior as the largest number of associated genes was found here at day0 and because AM is known for its central role in regulating emotions. Key genes and biological processes as identified from the lists of heat score associated genes are discussed next and linked to estrous behavioral expression, though the links are not always as expected.

### Genes and biological processes associated with estrous behavior in line with expectations

An association with heat scores was detected for the ***CGA ***gene, which encodes for the alpha subunit of glycoprotein hormones (FSH, LH, TSH), with associations being time and brain area specific. It seems that cows with clear expression of estrous behavior have relatively high expression of this gene in the hypothalamus around mid-cycle and low expression around start of estrus.

The ***OXT ***gene was found associated in the HC and DH whereas the ***AVP ***gene was found associated in the HC and AM. The known functional properties of these genes contributed to several enriched GO terms in the DAVID analysis, especially those related to female mating behavior. Oxytocin is released within the brain where it acts on specific oxytocin receptors to elicit effects like female sexual receptivity, grooming behavior and partner bonding [11]. In the presence of estrogen, oxytocin exerts an anxiolytic effect thereby favoring courtship and mating [12,13]. Similar to oxytocin, *AVP *is associated with sexual behavior and bonding [14] and its expression is under control of the steroids progesterone and estrogen [15,16]. In mice, the absence of estrogen receptors (ERα and ERβ) impairs social recognition similar to the effect of *OXT *gene deletion [17]. Studies with rodents and humans [18] demonstrate that oxytocin and vasopressin modulate complex socio-sexual behavior, typically under the influence of reproductive steroid hormones. The present association of *OXT *and *AVP *in several brain areas is in line with the above mentioned findings and suggests a major influence of these genes on estrous behavior expression in dairy cows.

The genes ***CCK*, *POMC, MCHR1, GABRA6, HTR2A ***and ***DRD2***, which associated with heat score in at least one brain area, are known to modulate emotional states like anxiety and satiety [19-21] or even sexual motivation. In sheep, dopamine-mediated D2 receptor (*DRD2*) signaling in the mediobasal hypothalamus affects female sexual motivation and receptivity [22]. Interactions between monoamines (dopamine, serotonin, noradrenaline) and steroid hormones play a major role in the integration of reproductive behavior and gonadal function [23]. The perception and awareness of male-related cues differs with the stage of estrous cycle, with releases of monoamines (linked to *HTR2A *and *DRD2) *and gamma-aminobutyric acid (GABA) (linked to *GABRA6*) in the mediobasal hypothalamus being triggered by such cues only when ewes are in estrus [24]. We found serotonin receptor 2A (*HTR2A*) associated with heat score at day12 in VH. Studies in female rats and hamsters have shown the inhibitory and facilitatory effects of serotonin receptor agonists and antagonists on the hypothalamic regulation of sexual receptivity [25,26], and this regulation is also mediated by GABAergic neurons interacting with serotonin containing neurons [27].

Noteworthy, are the heat score associated genes (***PTGDS, PTGIS, PTGFR*) **that regulate prostaglandin functioning. In the central nervous system, prostaglandins are involved in functions like thermoregulation and influencing neuronal morphology. Prostaglandins are also known to be under the influence of estradiol [28] and are capable of directly affecting neurons that synthesize and secrete gonadotropin-releasing hormone [29].

Another heat score associated gene, ***TTR***, a carrier of thyroid hormone, is known to influence anxiety [30], behavioral activity [31] and mental functions [32]. The melanin-concentrating hormone receptor, *MCHR1*, plays a role in metabolic rate and feed intake [33]. Changes in anxiety behavior and feeding motivation are likely to facilitate mating.

The present association between heat scores and the expression of ***ACHE ***and several cholinergic receptors **(*CHRM1, CHRM3 ***and ***CHRNA5) ***may be explained by the effect of the neurotransmitter, acetylcholine on arousal, plasticity and reward. The products of the muscarinic cholinergic receptor genes, *CHRM1 *and *CHRM3*, are G_q_-protein coupled receptors whose activation releases intracellular Ca^2+ ^via the phospholipase C - inositol 1,4,5-trisphosphate signaling pathway [34]. The genes for phospholipase C and inositol triphosphate kinase (***PLCB2, ITPKA***) and several protein kinases were also found associated to heat score. These findings may be explained based on the hypothesis put forward by Kow and Pfaff [35] that the membrane actions of estrogen can modulate the genomic actions of estrogen and that this transcriptional potentiation was mediated via signaling pathways requiring the activation of certain protein kinases and increased intracellular Ca^2+^.

### Genes and biological processes unexpectedly associated with estrous behavior

The finding of expected processes related to estrus as described in the previous section supports assumed neurophysiological mechanisms underlying female sexual behavior in dairy cows. Here, examples are given of more novel candidate genes and mechanisms. The ***TAC3 ***gene encodes the protein tachykinin (or neurokinin B), which in humans has been considered a critical regulator of gonadotropins (LH, FSH) via regulating GnRH secretion [36]. The present findings encourage further investigation towards the importance of tachykinin associated mechanisms in dairy cow fertility. Behavioral changes during estrus represent changes in central perception and processing of information, i.e. cognition, and some of the genes that associated with heat scores have been linked to cognition, e.g. ***PEBP1, MOBP, LTA4H *and *KCNN2***. *PEBP1 *has been suggested to be involved in chronic stress-induced memory impairment [37], *MOBP *has been linked to mood disorders [38], *LTA4H *to depression in women [39], and *KCNN2 *to anxiety and stress responses [40].

The gene ***LIPN1 ***in mice seems to establish a cross-talk between reproduction and metabolic events [41] and was here associated with heat scores. Also the ***POU1F1 ***gene, which encodes transcription factors involved in activation of growth hormone and prolactin, was found associated with heat score in the AP on day0. This gene may contribute in part to the generally observed reduction in estrous behavior in high producing cows. The heat score associated gene ***GARNL1 ***is noted here because it has been linked to egg productivity in laying hens [42] and, thus, may have a fertility related function.

A number of heat score associated genes have been linked to the immune system, e.g. ***CTLA4, IL1RL1, MARCO, FCRLA, IL33, CCL26 ***and ***CXCL10***, indicating the importance of cell-cell interactions. It has been shown that immunoglobulin superfamily proteins may play important roles in brain developmental processes and the functioning of neuronal networks in adults because they provide the ideal structure for protein-protein interactions and, thus, cell-cell interactions [43]. Remodeling of synaptic networks, which occurs during estrogen promoted female sexual behavior [7], may also be facilitated by immunoglobulins. Together with the many associated genes that are known to regulate cell fate, activity and morphology, this seems to underline the importance of neural tissue plasticity in the appropriate expression of estrous behavior.

The gene product Sodium/potassium-transporting ATPase subunit alpha-3 (***ATP1A3***), which was found to be associated to heat score at estrus in AP in our earlier study was also found in VH. *ATP1A3 *has been implicated in rapid-onset dystonia parkinsonism (RDP), characterized by sudden onset of neurological symptoms over hours to a few days [44], suggesting a role in the sudden onset of behavioral changes like during estrus.

### Estrous behavior associated genes expressed at estrus and mid-cycle

Including data from both day0 and day12 cows in a combined analysis not only revealed genes that were associated with estrous behavior regardless of phase of cycle but also resulted in greater statistical power and helped reveal some associated genes which could not be found in the separate analysis. For example, *DRD2 *(dopamine receptor D2) gene has been found associated in DH. However, care needs to be taken to interpret the results of the combined analysis where an associated gene was also found in one or both of the separate analyses. There could be cases where the day0+day12 combined analysis found an associated gene because of the effect of a strong association found in one of the separate analyses. An example of this case is *HTR2A *which did not associate with heat scores on day0, but did on day12 (R^2 ^= 0.27) and also weakly (R^2 ^= 0.1) in the day0+day12 combined analysis, probably as a carryover effect.

Interesting observations were also made by investigating associated genes that appeared in several brain areas and analyses. For example, the gene ***SLC17A7 ***found in VH at day0 and in the combined analysis and also in the AP, is known to mediate the uptake of glutamate into synaptic vesicles at presynaptic nerve terminals of excitatory neural cells and may also mediate the transport of inorganic phosphate [45]. The gene could be a contributor to neurotransmission associated with estrous behavior expression and was one of the genes contributing to several ion transport related GO terms found enriched in the DAVID analysis. The gene ***NKD1***, found in several brain areas across all analyses, was seen to contribute to several GO terms related to binding and signal transduction. It has been reported to have a role as an antagonist of Wnt signaling pathway which may influence the development of neurons in dorsal midbrain [46], suggesting again a link between neuronal plasticity and estrous behavior. The gene ***ANO8***, also found associated in several brain areas and analyses, is a member of the anoctamin family which has been implicated in calcium ion-activated chloride channels that perform several important functions including neuronal excitability.

**Figure 3:**
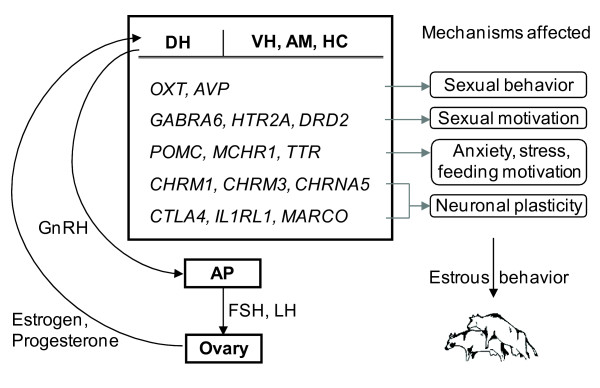
**Schematic representation of key genes found associated with estrous behavior and their relationship with estrus**. Some of the key genes whose expressions in the brain areas were found associated with estrous behavior are depicted here along with mechanisms affected during estrus that are important for estrous behavior. GnRH released from the DH stimulates release of FSH and LH from the AP which in turn influences the ovary to release estrogen and progesterone (Hypothalamic-pituitary-gonadal axis). In the brain areas, estrogen influences expression of *OXT *and *AVP*, which play major roles in the regulation of sexual behavior. The neurotransmitter receptors, *GABRA6, HTR2A and DRD2 *affect sexual motivation, whereas *CHRM1, CHRM3 and CHRNA5 *along with "immune related" genes like *CTLA4, IL1RL1 and MARCO *affect neuronal plasticity. The genes *POMC, MCHR1 and TTR *are involved in altering anxiety, stress and feeding motivation.

To summarize, several genes were found here whose expression levels in the brain areas were associated with the degree to which cows express estrous behavior. For some of these genes, there is a known function relating them to processes regulating estrous behavior, while for others, the current association suggests such a relation. We propose that the genes *OXT *and *AVP *play major roles in regulating estrous behavior along with genes affecting neurotransmission and neuronal plasticity. Genes, whose expression in mid-cycle associated with estrous behavior, may contribute to preparing the cow for the next estrus. Figure 3 depicts the key findings. This study may assist in the search for biomarkers for estrus detection or in screening for the most likely genes within QTLs associated with fertility. Further research with more animals sampled at multiple time points in the estrous cycle may improve our understanding of the dynamic regulation of estrous behavior over the cycle. Nevertheless, this first study provides an understanding of some of the genes and processes related to expression of estrous behavior in dairy cows.

## Conclusions

The study predicted estrous behavior associated genes in the 4 brain areas at two time points of the estrous cycle. These included genes expected to be related to estrous behavior as they influence states like socio-sexual behavior, anxiety, stress and feeding motivation (*OXT, AVP, POMC, MCHR1*), but also genes whose association with estrous behavior is novel and warrants further investigation. Studying these genes and the processes they control improves our understanding of the genomic regulation of estrous behavior expression, ultimately leading to better management strategies or tools to improve or monitor reproductive performance.

## Methods

### Data recording, sample isolation and microarray hybridization

Estrous behavior was recorded in 28 healthy Holstein Friesian cows from 30 days in milk (DIM) onwards till their time of sacrifice which varied between 77 and 139 DIM. The estrous behavior recorded in these cows were quantified as heat scores and the scores from multiple consecutive cycles per cow were averaged to obtain the average heat score per cow [6]. Of the 28 cows, 14 were sacrificed at the start of their estrous cycle (day0) and 14 around mid cycle (day12). Following sacrifice, brain tissue samples were collected from 4 brain areas: amygdala (AM), hippocampus (HC), dorsal hypothalamus (DH) and ventral hypothalamus (VH). Since the brain areas are not clearly demarcated, a standardized system was used for positioning and dissecting the brain which ensured that the collected samples representing each brain area in all cows were identical. The procedure followed for collecting brain samples is described in detail in Additional file 4. The study was approved by the Animal Care and Ethics Committee of the Animal Sciences Group of Wageningen University and Research Centre, Lelystad.

RNA extracted from brain tissue samples were hybridized on Bovine 24K oligonucleotide (70-mer) microarrays designed and produced by the Bovine Oligonucleotide Microarray Consortium (BOMC), USA http://www.bovineoligo.org/. This array was amongst the few whole genome bovine spotted microarrays available at the time this experiment was performed in the year 2007-08. The choice of microarray technology over RNA sequencing, which was not a well established technology then, was appropriate for fulfilling the objectives of this study in a precise and cost effective manner. The procedures followed for RNA preparation and microarray hybridization were as described in our earlier study [6]. The purity of the total RNA was assessed using the A260/280 ratios given by NanoDrop spectrophotometer and found to have a ratio above 1.8 indicating good quality RNA. In addition, we also performed agarose gel electrophoresis for a visual inspection of the RNA integrity. There was no indication of degraded RNA as deduced from the intensities of the 28S and 18S bands. Based on the combination of results of NanoDrop and gel electrophoresis, we were satisfied with the quality of RNA extracted by our extraction procedure. Five μg RNA was used per labelling using the RNA MICROMAX TSA labelling and detection kit (Perkin-Elmer). A total of 224 arrays (i.e. 28 cows × 4 brain areas × 2 dye swaps) were prepared in a common reference design with the dye labels swapped between individual samples from each brain area and a reference sample consisting of equal proportions of RNA from all four brain areas as well as AP of all cows. Processed slides were scanned using GenePix 4200A (Molecular Devices), with identical settings and the images processed using GenePix Pro 6 software (Molecular Devices). Regarding microarray quality and validation, a recent re-annotation of the probes in this array following the drafting of the bovine genome ensured that only good quality probes were considered in further analysis (details in the second following section). All array hybridizations and processing were done in a standard lab with experienced personnel using a standard microarray platform. Moreover, the experimental design used 14 biological replicates per time point which is relatively large and sufficient for a sound statistical analysis and the use of dye swap technical replication also improves the analysis. Given the above facts, we believe that a PCR validation of the individual probes was not required, the more so because we analysed clusters of genes instead of individual genes.

The array experiment data is publicly accessible on ArrayExpress (accession: E-TABM-916 on http://www.ebi.ac.uk/arrayexpress).

### Microarray data analysis and identification of estrous behavior associated genes

Microarray data pre-processing and analysis was done using the LIMMA (Linear models for microarray data) package [47] within Bioconductor project [48] of R statistical programming language http://www.r-project.org, identical to the procedures described and used in our earlier study [6]. Gene expression levels expressed as M-values (log differential-expression ratio of sample versus reference) were thus obtained for each brain area per cow. For each gene, we then modeled its expression level across all cows as a function of the cows' average heat score. For this we used the Bayesian hierarchical mixed model developed by Jia *et al*. [49] that employs orthogonal polynomials to quantify linear and non-linear associations between quantitative phenotypes and gene expression data. We used the algorithm of Jia *et al*. [49] coded in SAS^® ^language which the authors kindly provided. The program was run on SAS^® ^software, Version 9.1 of the SAS^® ^System for Windows. Similar to the approach in our previous study on the AP [6], here we used the SAS program for three separate analyses on data from each of the 4 brain areas to achieve the objectives of this study (summarized in Table 2). However, in contrast to the procedure followed in our earlier study, where gene expression was modeled as a function of average heat score with third order polynomials, here we used second order polynomials. The Bayesian hierarchical mixed model used here remains the same as was explained in our earlier study [6]. However, we limited our analyses to two orders, linear and quadratic, because the resulting relationships for third order polynomials were observed to be less reliable considering the low number of animals used in this study. For each gene, posterior probabilities were obtained for the two regression coefficients to be different from zero. Genes were considered to have a significant association when the posterior probability of at least one of the regression coefficients was larger than 0.80, thereby limiting the FDR to below 1% [50]. We performed the Gibbs sampling chains multiple times (arbitrarily set to 5 chains with each chain running 10,000 iterations of which 5000 were burn-in) for each analysis of a particular brain area and only those genes that appeared significant in all chains were considered in the functional analysis (see next section). By doing so, we limited the variations due to Gibbs sampling.

### Functional analysis of estrous behavior associated genes

The original annotation of the bovine 24 K oligonucleotide microarray provided by BOMC dates back to June 2007. For our analysis, we used the bovine oligonucleotide array probe re-annotation (Version 5) based on Ensembl http://www.ensembl.org release 56 (October 2009) provided on the EADGENE website by the authors of the oligo-set re-annotation pipeline, sigReannot [51]. For the re-annotation, out of the 23,496 probes (all control probes removed) on the bovine oligonucleotide array, only 16,620 probes that were assigned a quality score between 1 and 4 based on their specificity to hits on the bovine genome were considered. Probes with quality scores between 5 and 7 had either no hits or multiple hits and were not annotated as they were not specific.

For gaining insight into biological processes underlying estrous behavior, we performed functional analyses of the sets of estrous behavior associated genes (study sets) identified in the 3 analyses for the 4 brain areas and the anterior pituitary. To increase the accuracy of functional analysis, we used the re-annotated probes information. The probe re-annotation also provides the Ensembl gene ID of the orthologous human genes for 14,585 probes, which we used in the functional analysis instead of bovine genes so as to benefit from the greater gene annotation information available for human species.

Functional analysis was done using DAVID bioinformatics resources [52,53], a freely available web-based tool http://david.abcc.ncifcrf.gov/ that integrates biological data from several sources including GO [54] and biological pathway databases. In order to get an indication of the major processes over-represented among the annotated probes (genes) in the study sets (all linear/quadratic associated genes in each of the 3 analyses for the 4 brain areas), we interpreted the significance of the DAVID results based on EASE score (p value derived from a modified Fisher's exact test) [55] threshold set at 0.10. We did not consider multiple testing correction as it was too conservative for our purpose of discovery of biological themes in our study sets. Genes in the study sets were tested for enriched GO terms and KEGG [56] pathway terms using DAVID functional annotation tool. The population set against which the study set genes were tested consisted of the Ensembl IDs of the orthologous human genes, of which 11,589 remained after removing duplicates..

## List of abbreviations

AM: Amygdala; AP: Anterior Pituitary; BOMC: Bovine Oligonucleotide Microarray Consortium; DAVID: Database for Annotation, Visualization and Integrated Discovery; DH: Dorsal Hypothalamus, EADGENE: European Animal Disease Genomics Network of Excellence for Animal Health and Food Safety; FDR: False Discovery Rate; FSH: Follicle stimulating hormone; GnRH: Gonadotropin-releasing hormone; GO: Gene Ontology; HC: Hippocampus; KEGG: Kyoto Encyclopedia of Genes and Genomes; LH: Luteinizing hormone; QTL: Quantitative trait loci; SD: Standard Deviation; TSH: Thyroid stimulating hormone; VH: Ventral Hypothalamus;

## Authors' contributions

AACW performed the microarray experiments. AK performed the data analysis and drafted the manuscript. HAM and RFV helped with the statistical analysis. BB, HW, MFWP and MAS helped with the biological interpretation of the results. All authors read and helped in improving the manuscript.

## Supplementary Material

Additional file 1 **Association patterns between gene expression and heat score of estrous behavior associated genes identified in the four brain areas and anterior pituitary**. See legend at end of table. Click here for file

Additional file 2 **Venn diagrams showing the number of overlapping probes between the different brain areas per analysis**. The figures in brackets represent the total number of estrous behavior associated probes found in each brain area per analysis. Here, AM - Amygdala; HC - Hippocampus; DH - Dorsal Hypothalamus; VH - Ventral Hypothalamus; AP - Anterior Pituitary. Click here for file

Additional file 3 **Significantly enriched Gene Ontology and KEGG pathway terms in the estrous behavior associated gene lists**. Table shows all terms with p < 0.10 and includes gene ontology terms in the 3 categories: Biological Process, Molecular function and Cellular component. Click here for file

Additional file 4 **Collection of brain samples and pituitary from the experimental cows**. Procedure followed for collection of anterior pituitary and brain samples: amygdala, hippocampus, dorsal hypothalamus and ventral hypothalamus (with pictures). Click here for file

## References

[B1] Butler WR Nutritional interactions with reproductive performance in dairy cattle Anim Reprod Sci 2000 60-61 449 457 10.1016/S0378-4320(00)00076-2 10844215

[B2] Butler WR Energy balance relationships with follicular development, ovulation and fertility in postpartum dairy cows Livest Prod Sci 2003 83 2-3 211 218 10.1016/S0301-6226(03)00112-X

[B3] Veerkamp RF Beerda B Genetics and genomics to improve fertility in high producing dairy cows Theriogenology 2007 68 Supplement 1 S266 S273 17531307 10.1016/j.theriogenology.2007.04.034

[B4] Lucy MC Reproductive Loss in High-Producing Dairy Cattle: Where Will It End? J Dairy Sci 2001 84 6 1277 1293 10.3168/jds.S0022-0302(01)70158-0 11417685

[B5] Lopez H Satter LD Wiltbank MC Relationship between level of milk production and estrous behavior of lactating dairy cows Anim Reprod Sci 2004 81 3-4 209 223 10.1016/j.anireprosci.2003.10.009 14998648

[B6] Kommadath A Mulder HA de Wit AAC Woelders H Smits MA Beerda B Veerkamp RF Frijters ACJ te Pas MFW Gene expression patterns in anterior pituitary associated with quantitative measure of oestrous behaviour in dairy cows Animal 2010 4 08 1297 1307 10.1017/S1751731110000303 22444649

[B7] Pfaff D Hormone-driven mechanisms in the central nervous system facilitate the analysis of mammalian behaviours J Endocrinol 2005 184 3 447 453 10.1677/joe.1.05897 15749804

[B8] Mong JA Pfaff DW Hormonal symphony: steroid orchestration of gene modules for sociosexual behaviors Mol Psychiatry 2004 9 6 550 556 10.1038/sj.mp.4001493 15164085

[B9] Gallagher M Chiba AA The amygdala and emotion Curr Opin Neurobiol 1996 6 2 221 227 10.1016/S0959-4388(96)80076-6 8725964

[B10] Salamon E Esch T Stefano GB Role of amygdala in mediating sexual and emotional behavior via coupled nitric oxide release Acta Pharmacol Sin 2005 26 4 389 395 10.1111/j.1745-7254.2005.00083.x 15780186

[B11] Leng G Meddle SL Douglas AJ Oxytocin and the maternal brain Curr Opin Pharmacol 2008 8 6 731 734 10.1016/j.coph.2008.07.001 18656552

[B12] McCarthy MM McDonald CH Brooks PJ Goldman D An Anxiolytic Action of Oxytocin is Enhanced by Estrogen in the Mouse Physiol Behav 1997 60 5 1209 1215 10.1016/S0031-9384(96)00212-0 8916173

[B13] Mong JA Pfaff DW Hormonal symphony: steroid orchestration of gene modules for sociosexual behaviors Mol Psychiatry 9 6 550 556 10.1038/sj.mp.4001493 15164085

[B14] Curley JP Keverne EB Genes, brains and mammalian social bonds Trends Ecol Evol 2005 20 10 561 567 10.1016/j.tree.2005.05.018 16701435

[B15] Kalamatianos T Kalló I Goubillon ML Coen CW Cellular Expression of V1a Vasopressin Receptor mRNA in the Female Rat Preoptic Area: Effects of Oestrogen J Neuroendocrinol 2004 16 6 525 533 10.1111/j.1365-2826.2004.01199.x 15189327

[B16] Patisaul HB Scordalakes EM Young LJ Rissman EF Oxytocin, But Not Oxytocin Receptor, is Regulated by Oestrogen Receptor β; in the Female Mouse Hypothalamus J Neuroendocrinol 2003 15 8 787 793 10.1046/j.1365-2826.2003.01061.x 12834440

[B17] Choleris E Gustafsson JA Korach KS Muglia LJ Pfaff DW Ogawa S An estrogen-dependent four-gene micronet regulating social recognition: A study with oxytocin and estrogen receptor-α and -β knockout mice Proc Natl Acad Sci USA 2003 100 10 6192 6197 10.1073/pnas.0631699100 12730370 PMC156348

[B18] Donaldson ZR Young LJ Oxytocin, Vasopressin, and the Neurogenetics of Sociality Science 2008 322 5903 900 904 10.1126/science.1158668 18988842

[B19] Uhart M McCaul ME Oswald LM Choi L Wand GS GABRA6 gene polymorphism and an attenuated stress response Mol Psychiatry 2004 9 11 998 1006 10.1038/sj.mp.4001535 15197399

[B20] Rex A Marsden CA Fink H Cortical 5-HT-CCK interactions and anxiety-related behaviour of guinea-pigs: a microdialysis study Neurosci Lett 1997 228 2 79 82 10.1016/S0304-3940(97)00371-6 9209103

[B21] Millington G The role of proopiomelanocortin (POMC) neurones in feeding behaviour Nutrition & Metabolism 2007 4 1 18 17764572 10.1186/1743-7075-4-18 PMC2018708

[B22] Fabre-Nys C Chesneau D De La Riva C Hinton MR Locatelli A Ohkura S Kendrick KM Biphasic role of dopamine on female sexual behaviour via D2 receptors in the mediobasal hypothalamus Neuropharmacology 2003 44 354 366 10.1016/S0028-3908(02)00410-0 12604086

[B23] Fabre-Nys C Steroid control of monoamines in relation to sexual behaviour Rev Reprod 1998 3 1 31 41 10.1530/ror.0.0030031 9509987

[B24] Fabre-Nys C Ohkura S Kendrick KM Male Faces and Odours Evoke Differential Patterns of Neurochemical Release in the Mediobasal Hypothalamus of the Ewe During Oestrus: An Insight Into Sexual Motivation Eur J Neurosci 1997 9 1666 1677 10.1111/j.1460-9568.1997.tb01524.x 9283821

[B25] Uphouse L Female gonadal hormones, serotonin, and sexual receptivity Brain Res Rev 2000 33 2-3 242 257 10.1016/S0165-0173(00)00032-1 11011068

[B26] Caldwell HK Albers HE The Effects of Serotonin Agonists on the Hypothalamic Regulation of Sexual Receptivity in Syrian Hamsters Horm Behav 2002 42 1 78 84 10.1006/hbeh.2002.1803 12191650

[B27] Luine VN Wu V Hoffman CS Renner KJ GABAergic Regulation of Lordosis: Influence of Gonadal Hormones on Turnover of GABA and Interaction of GABA with 5-HT Neuroendocrinology 1999 69 6 438 445 10.1159/000054447 10364696

[B28] Amateau SK McCarthy MM A Novel Mechanism of Dendritic Spine Plasticity Involving Estradiol Induction of Prostaglandin-E2 J Neurosci 2002 22 19 8586 8596 12351732 10.1523/JNEUROSCI.22-19-08586.2002 PMC6757802

[B29] Jasoni CL Todman MG Han SK Herbison AE Expression of mRNAs Encoding Receptors That Mediate Stress Signals in Gonadotropin-Releasing Hormone Neurons of the Mouse Neuroendocrinology 2005 82 5-6 320 328 10.1159/000093155 16721036

[B30] Sousa JC Grandela C Fernández-Ruiz J Miguel Rd Sousa Ld Magalhães AI Saraiva MJ Sousa N Palha JA Transthyretin is involved in depression-like behaviour and exploratory activity J Neurochem 2004 88 5 1052 1058 10.1046/j.1471-4159.2003.02309.x 15009661

[B31] Venero C Guadano-Ferraz A Herrero AI Nordstrom K Manzano J de Escobar GM Bernal J Vennstrom B Anxiety, memory impairment, and locomotor dysfunction caused by a mutant thyroid hormone receptor α1 can be ameliorated by T3 treatment Genes Dev 2005 19 18 2152 2163 10.1101/gad.346105 16131613 PMC1221886

[B32] Nunez J Celi FS Ng L Forrest D Multigenic control of thyroid hormone functions in the nervous system Mol Cell Endocrinol 2008 287 1-2 1 12 10.1016/j.mce.2008.03.006 18448240 PMC2486256

[B33] Marsh DJ Weingarth DT Novi DE Chen HY Trumbauer ME Chen AS Guan XM Jiang MM Feng Y Camacho RE Melanin-concentrating hormone 1 receptor-deficient mice are lean, hyperactive, and hyperphagic and have altered metabolism Proc Natl Acad Sci USA 2002 99 5 3240 3245 10.1073/pnas.052706899 11867747 PMC122503

[B34] Billups D Billups B Challiss RAJ Nahorski SR Modulation of Gq-Protein-Coupled Inositol Trisphosphate and Ca2+ Signaling by the Membrane Potential J Neurosci 2006 26 39 9983 9995 10.1523/JNEUROSCI.2773-06.2006 17005862 PMC2266565

[B35] Kow LM Pfaff DW The membrane actions of estrogens can potentiate their lordosis behavior-facilitating genomic actions Proc Natl Acad Sci USA 2004 101 33 12354 12357 10.1073/pnas.0404889101 15302933 PMC514479

[B36] Topaloglu AK Reimann F Guclu M Yalin AS Kotan LD Porter KM Serin A Mungan NO Cook JR Ozbek MN TAC3 and TACR3 mutations in familial hypogonadotropic hypogonadism reveal a key role for Neurokinin B in the central control of reproduction Nat Genet 2009 41 3 354 358 10.1038/ng.306 19079066 PMC4312696

[B37] Feldmann RE Maurer MH Hunzinger C Lewicka S Buergers HF Kalenka A Hinkelbein J Broemme JO Seidler GH Martin E Reduction in rat phosphatidylethanolamine binding protein-1 (PEBP1) after chronic corticosterone treatment may be paralleled by cognitive impairment: A first study Stress: The International Journal on the Biology of Stress 2008 11 2 134 147 10.1080/10253890701649904 18311602

[B38] Sokolov BP Oligodendroglial abnormalities in schizophrenia, mood disorders and substance abuse. Comorbidity, shared traits, or molecular phenocopies? The International Journal of Neuropsychopharmacology 2007 10 04 547 555 10.1017/S1461145706007322 17291372

[B39] Zhao J Quyyumi AA Patel R Zafari AM Veledar E Onufrak S Shallenberger LH Jones L Vaccarino V Sex-Specific Association of Depression and a Haplotype in Leukotriene A4 Hydrolase Gene Psychosom Med 2009 71 7 691 696 10.1097/PSY.0b013e3181b05c57 19622707 PMC3113512

[B40] Mitra R Ferguson D Sapolsky RM SK2 potassium channel overexpression in basolateral amygdala reduces anxiety, stress-induced corticosterone secretion and dendritic arborization Mol Psychiatry 2009 14 9 847 855 10.1038/mp.2009.9 19204724 PMC2763614

[B41] Nadra K Charles A-SdP Medard JJ Hendriks WT Han GS Gres S Carman GM Saulnier-Blache JS Verheijen MHG Chrast R Phosphatidic acid mediates demyelination in Lpin1 mutant mice Genes Dev 2008 22 12 1647 1661 10.1101/gad.1638008 18559480 PMC2428062

[B42] Chih-Feng C Yow-Ling S Cheng-Ju Y Pin-Chi T Hui-Chiu C Yen-Pai L Laying traits and underlying transcripts, expressed in the hypothalamus and pituitary gland, that were associated with egg production variability in chickens Theriogenology 2007 68 9 1305 1315 10.1016/j.theriogenology.2007.08.032 17931698

[B43] Rougon G Hobert O New insights into the diversity and function of neuronal immunoglobulin superfamily molecules Annu Rev Neurosci 2003 26 1 207 238 10.1146/annurev.neuro.26.041002.131014 12598678

[B44] de Carvalho Aguiar P Sweadner KJ Penniston JT Zaremba J Liu L Caton M Linazasoro G Borg M Tijssen MAJ Bressman SB Mutations in the Na+/K+-ATPase [alpha]3 Gene ATP1A3 Are Associated with Rapid-Onset Dystonia Parkinsonism Neuron 2004 43 2 169 175 10.1016/j.neuron.2004.06.028 15260953

[B45] Aihara Y Mashima H Onda H Hisano S Kasuya H Hori T Yamada S Tomura H Yamada Y Inoue I Molecular Cloning of a Novel Brain-Type Na+-Dependent Inorganic Phosphate Cotransporter J Neurochem 2000 74 6 2622 2625 10820226 10.1046/j.1471-4159.2000.0742622.x

[B46] Chittka A Volff J Wizenmann A Identification of genes differentially expressed in dorsal and ventral chick midbrain during early Development BMC Dev Biol 2009 9 1 29 10.1186/1471-213X-9-29 19397791 PMC2686707

[B47] Smyth GK Gentleman R, Carey V, Dudoit S, Irizarry R, Huber W Limma: linear models for microarray data Bioinformatics and computational biology solutions using R and Bioconductor 2005 New York: Springer 397 420

[B48] Gentleman R Carey V Bates D Bolstad B Dettling M Dudoit S Ellis B Gautier L Ge Y Gentry J Bioconductor: open software development for computational biology and bioinformatics Genome Biology 2004 5 10 R80 10.1186/gb-2004-5-10-r80 15461798 PMC545600

[B49] Jia Z Tang S Mercola D Xu S Marchiori E, Moore JH Detection of quantitative trait associated genes using cluster analysis Evolutionary computation, machine learning and data mining in bioinformatics 2008 4973/2008 Berlin Heidelberg: Springer-Verlag 83 94

[B50] Jia Z Xu S Mapping quantitative trait loci for expression abundance Genetics 2007 176 1 611 623 10.1534/genetics.106.065599 17339210 PMC1893048

[B51] Casel P Moreews F Lagarrigue S Klopp C sigReannot: an oligo-set re-annotation pipeline based on similarities with the Ensembl transcripts and Unigene clusters BMC Proceedings 2009 3 Suppl 4 S3 10.1186/1753-6561-3-s4-s3 19615116 PMC2712746

[B52] Dennis G Sherman B Hosack D Yang J Gao W Lane HC Lempicki R DAVID: Database for Annotation, Visualization, and Integrated Discovery Genome Biology 2003 4 5 P3 10.1186/gb-2003-4-5-p3 12734009 PMC193660

[B53] Huang DW Sherman BT Lempicki RA Systematic and integrative analysis of large gene lists using DAVID bioinformatics resources Nature Protocols 2009 4 1 44 57 19131956 10.1038/nprot.2008.211

[B54] Ashburner M Ball CA Blake JA Botstein D Butler H Cherry JM Davis AP Dolinski K Dwight SS Eppig JT Gene Ontology: tool for the unification of biology Nat Genet 2000 25 1 25 29 10.1038/75556 10802651 PMC3037419

[B55] Hosack DA Dennis G Sherman BT Lane HC Lempicki RA Identifying biological themes within lists of genes with EASE Genome Biology 2003 4 10 R70 10.1186/gb-2003-4-10-r70 14519205 PMC328459

[B56] Kanehisa M Goto S KEGG: Kyoto Encyclopedia of Genes and Genomes Nucleic Acids Res 2000 28 27 30 10.1093/nar/28.1.27 10592173 PMC102409

