# Supplementary material for: Gene expression patterns in four brain areas associate with quantitative measure of estrous behavior in dairy cows
Source: BMC Genomics. 2011 Apr 19;12:200. doi: 10.1186/1471-2164-12-200 (PMC3110153; doi:10.1186/1471-2164-12-200)
Supplement: Additional file 1 — Association patterns between gene expression and heat score of estrous behavior associated genes identified in the four brain areas and anterior pituitary. See legend at end of table. [file 1471-2164-12-200-S1.PDF]

Association patterns between gene expression and heat score of estrous behaviour associated genes identified in the 4 brain areas and anterior pituitary *

Nr.	Gene Symbol	Gene Name	day0 analysis					day12 analysis					day0+day12 analysis				
			AM	HC	DH	VH	AP	AM	HC	DH	VH	AP	AM	HC	DH	VH	AP
1	A2ML1	alpha-2-macroglobulin-like 1					^										
2	AC006276.2														v		
3	AC069154.2									^							
4	AC107883.1											+					
5	AC135457.2		-														
6	ACADL	acyl-Coenzyme A dehydrogenase, long chain									^					^	
7	ACAP2	ArfGAP with coiled-coil, ankyrin repeat and PH domains 2														^	
8	ACHE	acetylcholinesterase (Yt blood group)							-								
9	AGT	angiotensinogen (serpin peptidase inhibitor, clade A, member 8)										v					
10	AHSA2	AHA1, activator of heat shock 90kDa protein ATPase homolog 2 (yeast)							v								
11	AIF1L	allograft inflammatory factor 1-like										v					
12	AKAP5	A kinase (PRKA) anchor protein 5									^						
13	AKT1	v-akt murine thymoma viral oncogene homolog 1	v										v				
14	AKT3	v-akt murine thymoma viral oncogene homolog 3 (protein kinase B, gamma)									^					^	
15	ALDH1A1	aldehyde dehydrogenase 1 family, member A1								v							
16	AMMECR1	Alport syndrome, mental retardation, midface hypoplasia and elliptocytosis chromosomal region gene 1													+		
17	ANGPTL7	angiopoietin-like 7										v					
18	ANKRD11	ankyrin repeat domain 11														v	
19	ANO8	anoctamin 8					v			v	v					v	
20	AP000843.1						^										
21	APOBEC1	apolipoprotein B mRNA editing enzyme, catalytic polypeptide 1							v								
22	ARGFX	arginine-fifty homeobox						^					^				
23	ATP1A2	ATPase, Na+/K+ transporting, alpha 2 (+) polypeptide					^										
24	ATP1A3	ATPase, Na+/K+ transporting, alpha 3 polypeptide					^				^						
25	ATP1B1	ATPase, Na+/K+ transporting, beta 1 polypeptide	v														
26	ATP2A1	ATPase, Ca++ transporting, cardiac muscle, fast twitch 1			^												
27	AVIL	advillin													^		
28	AVP	arginine vasopressin	-	-						v				-			
29	BAIAP3	BAI1-associated protein 3		-													
30	BDP1	B double prime 1, subunit of RNA polymerase III transcription initiation factor IIIB	-														
31	BNIP2	BCL2/adenovirus E1B 19kDa interacting protein 2							^								
32	BST2	bone marrow stromal cell antigen 2														^	
33	C11orf66	chromosome 11 open reading frame 66													-		

Nr.	Gene Symbol	Gene Name	day0 analysis					day12 analysis					day0+day12 analysis				
			AM	HC	DH	VH	AP	AM	HC	DH	VH	AP	AM	HC	DH	VH	AP
34	C13orf34	chromosome 13 open reading frame 34									v						
35	C14orf104	chromosome 14 open reading frame 104									^					^	
36	C14orf50	chromosome 14 open reading frame 50				^						^					
37	C17orf48	chromosome 17 open reading frame 48	-														
38	C1orf9	chromosome 1 open reading frame 9	-														
39	C20orf201	chromosome 20 open reading frame 201														v	
40	C21orf62	chromosome 21 open reading frame 62														^	
41	C21orf63	chromosome 21 open reading frame 63								v							
42	C2orf65	chromosome 2 open reading frame 65						v					v				
43	C3orf41	chromosome 3 open reading frame 41									v					v	
44	C6orf204	chromosome 6 open reading frame 204									^						
45	C7orf53	chromosome 7 open reading frame 53						^									
46	C7orf63	chromosome 7 open reading frame 63							^								
47	C8orf47	chromosome 8 open reading frame 47							^								
48	C9orf142	chromosome 9 open reading frame 142				^		v									+
49	CA2	carbonic anhydrase II					^										
50	CACNA1D	calcium channel, voltage-dependent, L type, alpha 1D subunit			^												
51	CACNG6	calcium channel, voltage-dependent, gamma subunit 6														^	
52	CALCR	calcitonin receptor	-														
53	CALY	calcyon neuron-specific vesicular protein										^					
54	CASP5	caspase 5, apoptosis-related cysteine peptidase								v							
55	CCDC114	coiled-coil domain containing 114														^	
56	CCDC125	coiled-coil domain containing 125					+										
57	CCDC137	coiled-coil domain containing 137				^											
58	CCDC47	coiled-coil domain containing 47														-	
59	CCDC64B	coiled-coil domain containing 64B			v												
60	CCDC70	coiled-coil domain containing 70										^					
61	CCDC80	coiled-coil domain containing 80					^										
62	CCDC88B	coiled-coil domain containing 88B	-		v		v	+		^	^						
63	CCK	cholecystokinin								^							
64	CCL26	chemokine (C-C motif) ligand 26	^														
65	CD302	CD302 molecule														^	
66	CDC42BPA	CDC42 binding protein kinase alpha (DMPK-like)									^						
67	CDC42EP3	CDC42 effector protein (Rho GTPase binding) 3			v												

Nr.	Gene Symbol	Gene Name	day0 analysis					day12 analysis					day0+day12 analysis				
			AM	HC	DH	VH	AP	AM	HC	DH	VH	AP	AM	HC	DH	VH	AP
68	CDT1	chromatin licensing and DNA replication factor 1					v										
69	CGA	glycoprotein hormones, alpha polypeptide		^		-		v	-	^	^		v	-			
70	CHL1	cell adhesion molecule with homology to L1CAM (close homolog of L1)										^					
71	CHN1	chimerin (chimaerin) 1										^					
72	CHRM1	cholinergic receptor, muscarinic 1								v							
73	CHRM3	cholinergic receptor, muscarinic 3									^						
74	CHRNA5	cholinergic receptor, nicotinic, alpha 5	v														
75	CHST1	carbohydrate (keratan sulfate Gal-6) sulfotransferase 1					^					v					
76	CIAO1	cytosolic iron-sulfur protein assembly 1 homolog (S. cerevisiae)							^					^			
77	CLDN11	claudin 11					^										
78	CLEC14A	C-type lectin domain family 14, member A	-														
79	CLK1	CDC-like kinase 1	+														
80	COL11A1	collagen, type XI, alpha 1										-					
81	COL11A2	collagen, type XI, alpha 2														^	
82	COL1A1	collagen, type I, alpha 1														^	
83	CPNE1	copine I							^								
84	CRADD	CASP2 and RIPK1 domain containing adaptor with death domain									^						
85	CRB1	crumbs homolog 1 (Drosophila)										+					+
86	CRYL1	crystallin, lambda 1							+								
87	CRYM	crystallin, mu										v					
88	CTLA4	cytotoxic T-lymphocyte-associated protein 4	v			^					v		v				
89	CUL5	cullin 5														^	
90	CXCL10	chemokine (C-X-C motif) ligand 10										^					
91	CXCL14	chemokine (C-X-C motif) ligand 14										v					
92	CYP26A1	cytochrome P450, family 26, subfamily A, polypeptide 1			+												
93	DDX6	DEAD (Asp-Glu-Ala-Asp) box polypeptide 6									^						
94	DES	desmin									-						
95	DHPS	deoxyhypusine synthase									^						
96	DKK1	dickkopf homolog 1 (Xenopus laevis)			^												
97	DLGAP1	discs, large (Drosophila) homolog-associated protein 1				v											
98	DNAH11	dynein, axonemal, heavy chain 11	^														
99	DNAJC24	DnaJ (Hsp40) homolog, subfamily C, member 24									v						
100	DOK2	docking protein 2, 56kDa				^											
101	DOLPP1	dolichyl pyrophosphate phosphatase 1												+			

Nr.	Gene Symbol	Gene Name	day0 analysis					day12 analysis					day0+day12 analysis				
			AM	HC	DH	VH	AP	AM	HC	DH	VH	AP	AM	HC	DH	VH	AP
102	DRD2	dopamine receptor D2													-		
103	DSG4	desmoglein 4	-														
104	ECD	ecdysoneless homolog (Drosophila)						^			^		^			^	
105	EDN3	endothelin 3	-														
106	EFCAB7	EF-hand calcium binding domain 7	v														
107	EFR3A	EFR3 homolog A (S. cerevisiae)									v					v	
108	EGFLAM	EGF-like, fibronectin type III and laminin G domains													-		
109	EIF4G3	eukaryotic translation initiation factor 4 gamma, 3					v										
110	ENKUR	enkurin, TRPC channel interacting protein												^			
111	ENPP2	ectonucleotide pyrophosphatase/phosphodiesterase 2					v					v					v
112	ENTPD1	ectonucleoside triphosphate diphosphohydrolase 1									v						
113	ETV4	ets variant 4	-														
114	EVPL	envoplakin		^													
115	FAM131A	family with sequence similarity 131, member A					^										
116	FAM160A2	family with sequence similarity 160, member A2		-										-	^		
117	FAM168A	family with sequence similarity 168, member A									^						
118	FAM5C	family with sequence similarity 5, member C					^										
119	FAM71E1	family with sequence similarity 71, member E1	-														
120	FANCM	Fanconi anemia, complementation group M														^	
121	FBP2	fructose-1,6-bisphosphatase 2			-												
122	FCRLA	Fc receptor-like A						^					^				
123	FEM1C	fem-1 homolog c (C. elegans)													v		
124	FHAD1	forkhead-associated (FHA) phosphopeptide binding domain 1													v		
125	FILIP1	filamin A interacting protein 1				v											
126	FKBP5	FK506 binding protein 5							+								
127	FLT1	fms-related tyrosine kinase 1 (vascular endothelial growth factor/vascular permeability factor receptor)							+								
128	FNIP2	folliculin interacting protein 2						v					v				
129	FUS	fusion (involved in t(12;16) in malignant liposarcoma)														v	
130	FUT11	fucosyltransferase 11 (alpha (1,3) fucosyltransferase)	-														
131	FXC1	fracture callus 1 homolog (rat)	-														
132	GABRA6	gamma-aminobutyric acid (GABA) A receptor, alpha 6	^														
133	GALK2	galactokinase 2						^							v		
134	GARNL1	GTPase activating Rap/RanGAP domain-like 1									^						
135	GAS8	growth arrest-specific 8	v														

Nr.	Gene Symbol	Gene Name	day0 analysis					day12 analysis					day0+day12 analysis				
			AM	HC	DH	VH	AP	AM	HC	DH	VH	AP	AM	HC	DH	VH	AP
136	GINS1	GINS complex subunit 1 (Psf1 homolog)	v														
137	GLI2	GLI family zinc finger 2						-									
138	GLT8D3	glycosyltransferase 8 domain containing 3														^	
139	GMFB	glia maturation factor, beta							^					+			
140	GNA12	guanine nucleotide binding protein (G protein) alpha 12		^													
141	GNA15	guanine nucleotide binding protein (G protein), alpha 15 (Gq class)	v					-			v					v	
142	GNAS	GNAS complex locus			-												
143	GNB2L1	guanine nucleotide binding protein (G protein), beta polypeptide 2-like 1								-							
144	GPC3	glypican 3										v					
145	GPN2	GPN-loop GTPase 2							+								
146	GPR19	G protein-coupled receptor 19	-														
147	GPX3	glutathione peroxidase 3 (plasma)		-										-			
148	GRHL2	grainyhead-like 2 (Drosophila)				-	+			^						v	
149	GTF2E2	general transcription factor IIE, polypeptide 2, beta 34kDa														^	
150	HARBI1	harbinger transposase derived 1				-											
151	HEPACAM	hepatocyte cell adhesion molecule					^										
152	HIGD1B	HIG1 hypoxia inducible domain family, member 1B					v										
153	HIST1H1C	histone cluster 1, H1c										^					
154	HLTF	helicase-like transcription factor													^		
155	HPCA	hippocalcin								v							
156	HPDL	4-hydroxyphenylpyruvate dioxygenase-like	-														
157	HTR2A	5-hydroxytryptamine (serotonin) receptor 2A									^					^	
158	HTRA4	HtrA serine peptidase 4									^						
159	IKBKAP	inhibitor of kappa light polypeptide gene enhancer in B-cells, kinase complex-associated protein										^					
160	IL1RL1	interleukin 1 receptor-like 1						^					^				
161	IL33	interleukin 33										v					
162	INPP5F	inositol polyphosphate-5-phosphatase F	-								^						
163	ISL2	ISL LIM homeobox 2													^		
164	ISOC1	isochorismatase domain containing 1						v					v				
165	ITPKA	inositol 1,4,5-trisphosphate 3-kinase A								^	^						
166	KCNJ15	potassium inwardly-rectifying channel, subfamily J, member 15	-														
167	KCNN2	potassium intermediate/small conductance calcium-activated channel, subfamily N, member 2	^														
168	KIAA0586	KIAA0586	v														
169	KIAA1462	KIAA1462									^						

Nr.	Gene Symbol	Gene Name	day0 analysis					day12 analysis					day0+day12 analysis				
			AM	HC	DH	VH	AP	AM	HC	DH	VH	AP	AM	HC	DH	VH	AP
170	KIAA1543	KIAA1543									^					^	
171	KIAA1755	KIAA1755	-														
172	KIAA1967	KIAA1967								-							
173	KIF1A	kinesin family member 1A	-														
174	KIF27	kinesin family member 27														^	
175	KLC2	kinesin light chain 2									^					^	
176	KLHL28	kelch-like 28 (Drosophila)	-														
177	KNTC1	kinetochore associated 1													v		
178	LAMA2	laminin, alpha 2												-			
179	LAT	linker for activation of T cells		^				^	v	-	v			v	v	-	
180	LIFR	leukemia inhibitory factor receptor alpha									v					v	
181	LIG4	ligase IV, DNA, ATP-dependent				^											
182	LIN54	lin-54 homolog (C. elegans)								^							
183	LIPK	lipase, family member K				^											
184	LPGAT1	lysophosphatidylglycerol acyltransferase 1							+					+			
185	LPIN1	lipin 1														^	
186	LRCH2	leucine-rich repeats and calponin homology (CH) domain containing 2		^													
187	LRRC38	leucine rich repeat containing 38									v				^		
188	LRRC69	leucine rich repeat containing 69									^					^	
189	LRRC8D	leucine rich repeat containing 8 family, member D	-														
190	LRRK2	leucine-rich repeat kinase 2				-											
191	LTA4H	leukotriene A4 hydrolase	+														
192	LUM	lumican														-	
193	LXN	latexin										^					
194	LYST	lysosomal trafficking regulator													^		
195	MAK16	MAK16 homolog (S. cerevisiae)													^		
196	MAP3K15	mitogen-activated protein kinase kinase kinase 15								^							
197	MAP3K7IP3	mitogen-activated protein kinase kinase kinase 7 interacting protein 3									^						
198	MAPK13	mitogen-activated protein kinase 13													^		
199	MAPK8IP3	mitogen-activated protein kinase 8 interacting protein 3	v														
200	MARCO	macrophage receptor with collagenous structure				^										-	
201	MARK4	MAP/microtubule affinity-regulating kinase 4			-					^							
202	MATN2	matrilin 2									^						
203	MCHR1	melanin-concentrating hormone receptor 1	v														

Nr.	Gene Symbol	Gene Name	day0 analysis					day12 analysis					day0+day12 analysis				
			AM	HC	DH	VH	AP	AM	HC	DH	VH	AP	AM	HC	DH	VH	AP
204	MCOLN3	mucolipin 3					+										
205	MEGF9	multiple EGF-like-domains 9										^					
206	MKL1	megakaryoblastic leukemia (translocation) 1	v														
207	MKNK2	MAP kinase interacting serine/threonine kinase 2													^		
208	MLC1	megalencephalic leukoencephalopathy with subcortical cysts 1					^										
209	MME	membrane metallo-endopeptidase													^		
210	MOBKL2C	MOB1, Mps One Binder kinase activator-like 2C (yeast)					+				^						
211	MOBP	myelin-associated oligodendrocyte basic protein					^										
212	MOCOS	molybdenum cofactor sulfurase		-	v			^						v	^		
213	MOCS1	molybdenum cofactor synthesis 1	-														
214	MRAP2	melanocortin 2 receptor accessory protein 2					v										
215	MRPL35	mitochondrial ribosomal protein L35										+					
216	MTMR10	myotubularin related protein 10														^	
217	MTMR14	myotubularin related protein 14										^					
218	MYH10	myosin, heavy chain 10, non-muscle									^						
219	MYL9	myosin, light chain 9, regulatory	v														
220	NCF2	neutrophil cytosolic factor 2							+								
221	NDRG2	NDRG family member 2					^										
222	NDRG4	NDRG family member 4													^		
223	NDUFS1	NADH dehydrogenase (ubiquinone) Fe-S protein 1, 75kDa (NADH-coenzyme Q reductase)	v					v									
224	NEUROD2	neurogenic differentiation 2				v	^										
225	NGEF	neuronal guanine nucleotide exchange factor					^										
226	NKD1	naked cuticle homolog 1 (Drosophila)		^	v	v		v	v	-		+				v	v
227	NOL10	nucleolar protein 10									^						
228	NPHP4	nephronophthisis 4														^	
229	NR2E3	nuclear receptor subfamily 2, group E, member 3	+														
230	NR4A1	nuclear receptor subfamily 4, group A, member 1					+										
231	NRGN	neurogranin (protein kinase C substrate, RC3)										^					
232	NSDHL	NAD(P) dependent steroid dehydrogenase-like													v		
233	NUCKS1	nuclear casein kinase and cyclin-dependent kinase substrate 1							+								
234	NUF2	NUF2, NDC80 kinetochore complex component, homolog (S. cerevisiae)							+					+			
235	NUP107	nucleoporin 107kDa	^	^													
236	NXPH2	neurexophilin 2										+					
237	OPCML	opioid binding protein/cell adhesion molecule-like					^										

Nr.	Gene Symbol	Gene Name	day0 analysis					day12 analysis					day0+day12 analysis				
			AM	HC	DH	VH	AP	AM	HC	DH	VH	AP	AM	HC	DH	VH	AP
238	OTUB2	OTU domain, ubiquitin aldehyde binding 2		^													
239	OXT	oxytocin, prepropeptide		-	^					-				-			
240	PAK1	p21 protein (Cdc42/Rac)-activated kinase 1														v	
241	PCP4	Purkinje cell protein 4		-										-			
242	PCTK3	PCTAIRE protein kinase 3								^							
243	PDIA5	protein disulfide isomerase family A, member 5		-										-			
244	PEBP1	phosphatidylethanolamine binding protein 1			-												
245	PEG3AS	PEG3 antisense RNA (non-protein coding)														^	
246	PHKA1	phosphorylase kinase, alpha 1 (muscle)	+					v					v				
247	PHKB	phosphorylase kinase, beta							+							v	
248	PHTF2	putative homeodomain transcription factor 2							^								
249	PIK3C2B	phosphoinositide-3-kinase, class 2, beta polypeptide														^	
250	PIK3C2G	phosphoinositide-3-kinase, class 2, gamma polypeptide									^					^	
251	PKD2	polycystic kidney disease 2 (autosomal dominant)													^		
252	PLAT	plasminogen activator, tissue					^										
253	PLCB2	phospholipase C, beta 2	-														
254	PLD3	phospholipase D family, member 3															^
255	PLP2	proteolipid protein 2 (colonic epithelium-enriched)					-										
256	PLXNC1	plexin C1					v										
257	POLL	polymerase (DNA directed), lambda														v	
258	POMC	proopiomelanocortin		^													
259	POU1F1	POU class 1 homeobox 1					v										
260	PPPDE1	PPPDE peptidase domain containing 1														^	
261	PQLC1	PQ loop repeat containing 1	-														
262	PRCC	papillary renal cell carcinoma (translocation-associated)							^								
263	PRHOXNB	parahox cluster neighbor	+														
264	PRKCQ	protein kinase C, theta	-														
265	PSRC1	proline/serine-rich coiled-coil 1									v						
266	PTGDS	prostaglandin D2 synthase 21kDa (brain)										v					
267	PTGFR	prostaglandin F receptor (FP)							+								
268	PTGIS	prostaglandin I2 (prostacyclin) synthase											v				
269	PTPRZ1	protein tyrosine phosphatase, receptor-type, Z polypeptide 1										v					
270	PVRL4	poliovirus receptor-related 4	-														
271	RAB19	RAB19, member RAS oncogene family	+					v			v				v	v	

Nr.	Gene Symbol	Gene Name	day0 analysis					day12 analysis					day0+day12 analysis				
			AM	HC	DH	VH	AP	AM	HC	DH	VH	AP	AM	HC	DH	VH	AP
272	RASL10A	RAS-like, family 10, member A								^							
273	RBBP6	retinoblastoma binding protein 6									v						
274	RBM46	RNA binding motif protein 46							+								
275	REEP3	receptor accessory protein 3						v									
276	REPIN1	replication initiator 1													-		
277	RHBDF1	rhomboid 5 homolog 1 (Drosophila)				^										^	
278	RICTOR	RPTOR independent companion of MTOR, complex														^	
279	RNASE4	ribonuclease, RNase A family, 4				^	+				^				^	^	
280	RNF149	ring finger protein 149		^													
281	RNF175	ring finger protein 175													v		
282	RNF24	ring finger protein 24														^	
283	ROD1	ROD1 regulator of differentiation 1 (S. pombe)	v						^								
284	RP11-244H3.1								+								
285	RPL24	ribosomal protein L24												+			
286	RRP7A	ribosomal RNA processing 7 homolog A (S. cerevisiae)	-								^					^	
287	SAT1	spermidine/spermine N1-acetyltransferase 1					+										
288	SEP14	septin 14			^								v	^			
289	SERPINE2	serpin peptidase inhibitor, clade E (nexin, plasminogen activator inhibitor type 1), member 2										v					v
290	SIX6	SIX homeobox 6				-								-			
291	SLC13A4	solute carrier family 13 (sodium/sulfate symporters), member 4							^								
292	SLC16A7	solute carrier family 16, member 7 (monocarboxylic acid transporter 2)							^								
293	SLC17A7	solute carrier family 17 (sodium-dependent inorganic phosphate cotransporter), member 7				-						+				v	^
294	SLC1A3	solute carrier family 1 (glial high affinity glutamate transporter), member 3			-		^										
295	SLC29A1	solute carrier family 29 (nucleoside transporters), member 1									v						
296	SLC35B3	solute carrier family 35, member B3							^					+			
297	SLC38A11	solute carrier family 38, member 11								^							
298	SLC46A2	solute carrier family 46, member 2													v		
299	SLTM	SAFB-like, transcription modulator									^					^	
300	SMAD1	SMAD family member 1						^									
301	SMC1A	structural maintenance of chromosomes 1A		^													
302	SNRPA	small nuclear ribonucleoprotein polypeptide A														-	
303	SNX1	sorting nexin 1				^											
304	SNX27	sorting nexin family member 27	v														
305	SNX3	sorting nexin 3	^														

Nr.	Gene Symbol	Gene Name	day0 analysis					day12 analysis					day0+day12 analysis				
			AM	HC	DH	VH	AP	AM	HC	DH	VH	AP	AM	HC	DH	VH	AP
306	SNX31	sorting nexin 31													v		
307	SNX7	sorting nexin 7	-														
308	SPARCL1	SPARC-like 1 (hevin)				^											
309	SPON1	spondin 1, extracellular matrix protein										+			-		
310	SPOPL	speckle-type POZ protein-like													+		
311	SPTBN4	spectrin, beta, non-erythrocytic 4	v														
312	ST6GALNAC1	ST6 (alpha-N-acetyl-neuraminyl-2,3-beta-galactosyl-1,3)-N-acetylgalactosaminide alpha-2,6-sialyltransferase 1						v									
313	STMN2	stathmin-like 2					^					+					
314	STMN4	stathmin-like 4										v					
315	SULT4A1	sulfotransferase family 4A, member 1															+
316	SYN2	synapsin II					^										
317	SYNE2	spectrin repeat containing, nuclear envelope 2									^						
318	SYTL2	synaptotagmin-like 2					^										
319	TAC3	tachykinin 3					+										
320	TBC1D2	TBC1 domain family, member 2													v		
321	TCL1A	T-cell leukemia/lymphoma 1A		^	v	v	v	^	v	v	v				v	v	
322	TEP1	telomerase-associated protein 1	+					-			v				v	v	
323	TES	testis derived transcript (3 LIM domains)	-								^					^	
324	TF	transferrin										^					
325	THBD	thrombomodulin	v		v			^			^				v	^	
326	THBS1	thrombospondin 1					+										
327	TM4SF20	transmembrane 4 L six family member 20			v					+							
328	TM4SF4	transmembrane 4 L six family member 4	^														
329	TMEFF2	transmembrane protein with EGF-like and two follistatin-like domains 2															^
330	TMEM30B	transmembrane protein 30B												-			
331	TMEM35	transmembrane protein 35										^					
332	TMPRSS7	transmembrane protease, serine 7	v														
333	TNFRSF1B	tumor necrosis factor receptor superfamily, member 1B									^					^	
334	TNKS1BP1	tankyrase 1 binding protein 1, 182kDa														^	
335	TOM1L2	target of myb1-like 2 (chicken)	v														
336	TOP3B	topoisomerase (DNA) III beta									v						
337	TRADD	TNFRSF1A-associated via death domain	-														
338	TRAF3IP3	TRAF3 interacting protein 3														^	
339	TRPV3	transient receptor potential cation channel, subfamily V, member 3		^													

Nr.	Gene Symbol	Gene Name	day0 analysis					day12 analysis					day0+day12 analysis				
			AM	HC	DH	VH	AP	AM	HC	DH	VH	AP	AM	HC	DH	VH	AP
340	TSC22D2	TSC22 domain family, member 2	-														
341	TSEN54	tRNA splicing endonuclease 54 homolog (S. cerevisiae)													v		
342	TSPAN15	tetraspanin 15	-														
343	TSPAN9	tetraspanin 9	v														
344	TSPYL1	TSPY-like 1			v	^			+			+		+			
345	TTC17	tetratricopeptide repeat domain 17	-														
346	TTC25	tetratricopeptide repeat domain 25									^						
347	TTC35	tetratricopeptide repeat domain 35									^						
348	TTC38	tetratricopeptide repeat domain 38														^	
349	TTF1	transcription termination factor, RNA polymerase I	+														
350	TTR	transthyretin		^	v			v		v					v		
351	UGT8	UDP glycosyltransferase 8					^										
352	UNC13C	unc-13 homolog C (C. elegans)									^					^	
353	UNC5B	unc-5 homolog B (C. elegans)										^					^
354	USP1	ubiquitin specific peptidase 1							v							v	
355	VAT1L	vesicle amine transport protein 1 homolog (T. californica)-like					^										
356	VIM	vimentin										v					
357	WDR67	WD repeat domain 67							v								
358	WDR78	WD repeat domain 78	v														
359	WIF1	WNT inhibitory factor 1										v					
360	XRCC3	X-ray repair complementing defective repair in Chinese hamster cells 3								v							
361	XRCC4	X-ray repair complementing defective repair in Chinese hamster cells 4														^	
362	ZAK	sterile alpha motif and leucine zipper containing kinase AZK						^					^				
363	ZBTB10	zinc finger and BTB domain containing 10									^					^	
364	ZC3H18	zinc finger CCCH-type containing 18		-						^	v						
365	ZC3H7B	zinc finger CCCH-type containing 7B													-		
366	ZCCHC7	zinc finger, CCHC domain containing 7												+			
367	ZMYM1	zinc finger, MYM-type 1														^	
368	ZNF192	zinc finger protein 192		-													
369	ZNF449	zinc finger protein 449				^											
370	ZNF462	zinc finger protein 462														^	
371	ZNF512B	zinc finger protein 512B														^	
372	ZNF787	zinc finger protein 787	v														

Nr.	Gene Symbol	Gene Name	day0 analysis					day12 analysis					day0+day12 analysis				
			AM	HC	DH	VH	AP	AM	HC	DH	VH	AP	AM	HC	DH	VH	AP

* Association patterns between gene expression and heat score	
+	Positive linear
-	Negative linear
v	Positive quadratic
^	Negative quadratic

AM	Amygdala
HC	Hippocampus
DH	Dorsal hypothalamus
VH	Ventral hypothalamus
AP	Anterior pituitary
