# Supplementary figures and images for: Gene expression patterns in four brain areas associate with quantitative measure of estrous behavior in dairy cows
Source: BMC Genomics. 2011 Apr 19;12:200. doi: 10.1186/1471-2164-12-200 (PMC3110153; doi:10.1186/1471-2164-12-200)

### "day0" analysis

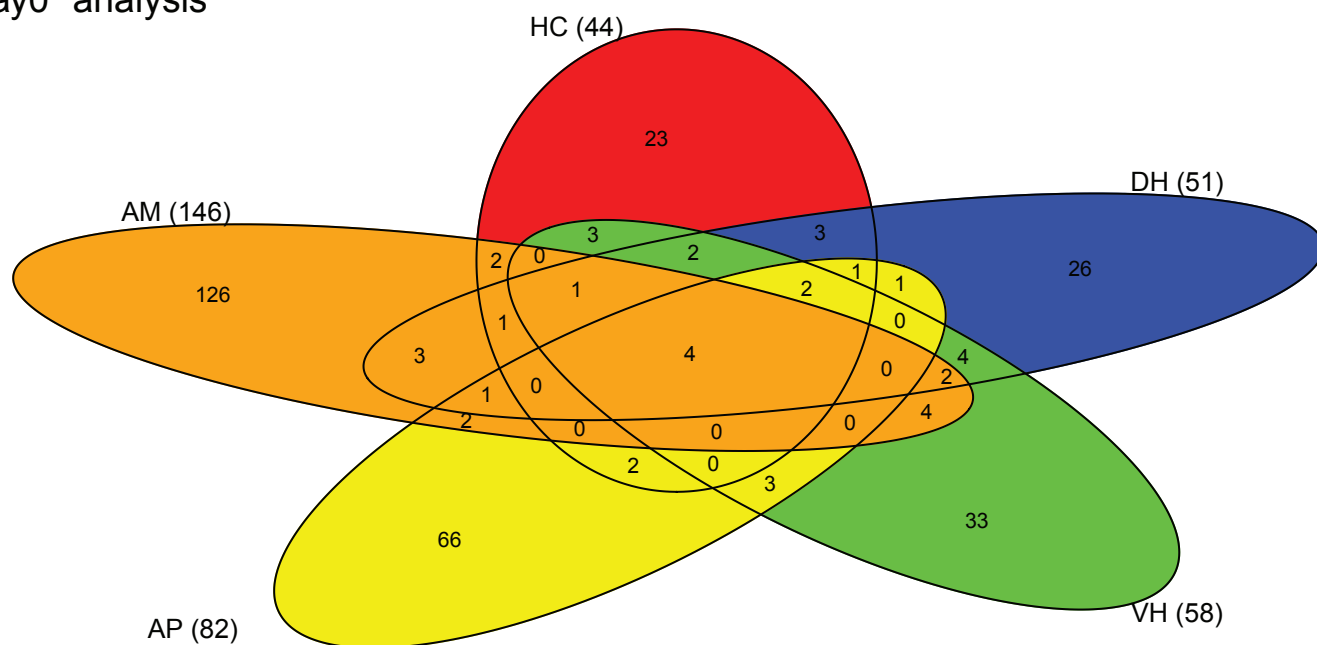

### "day12" analysis

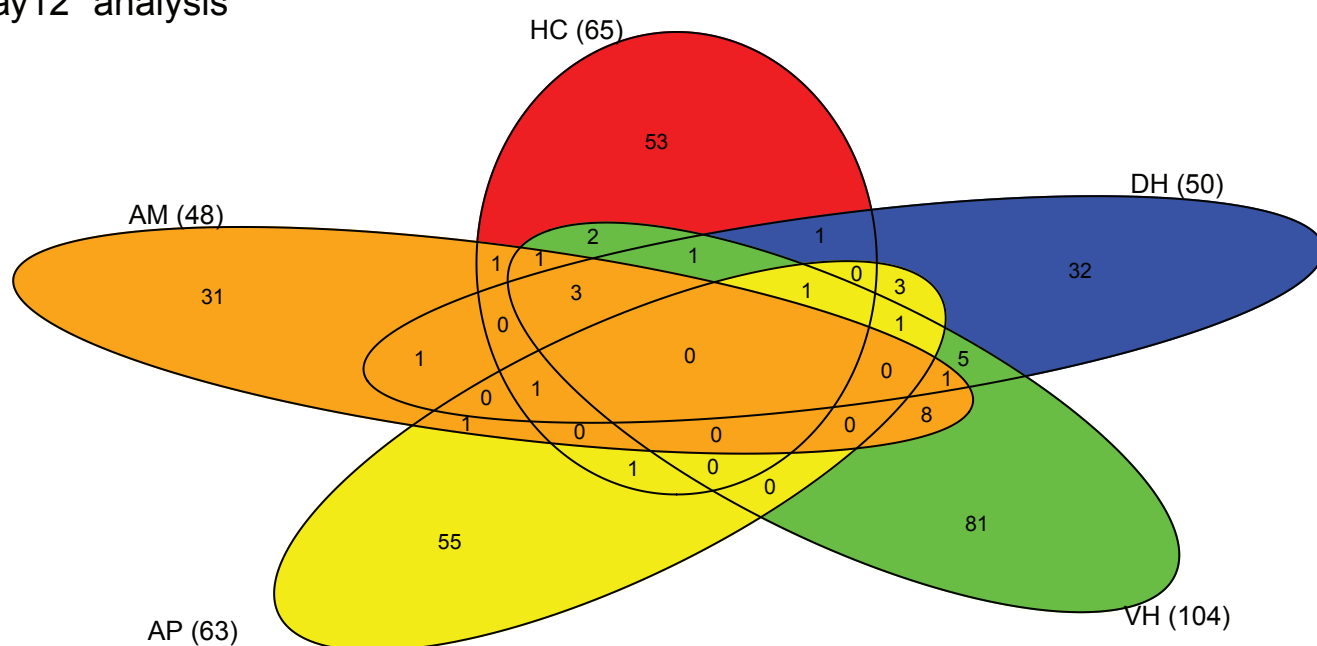

### "day0+day12" analysis

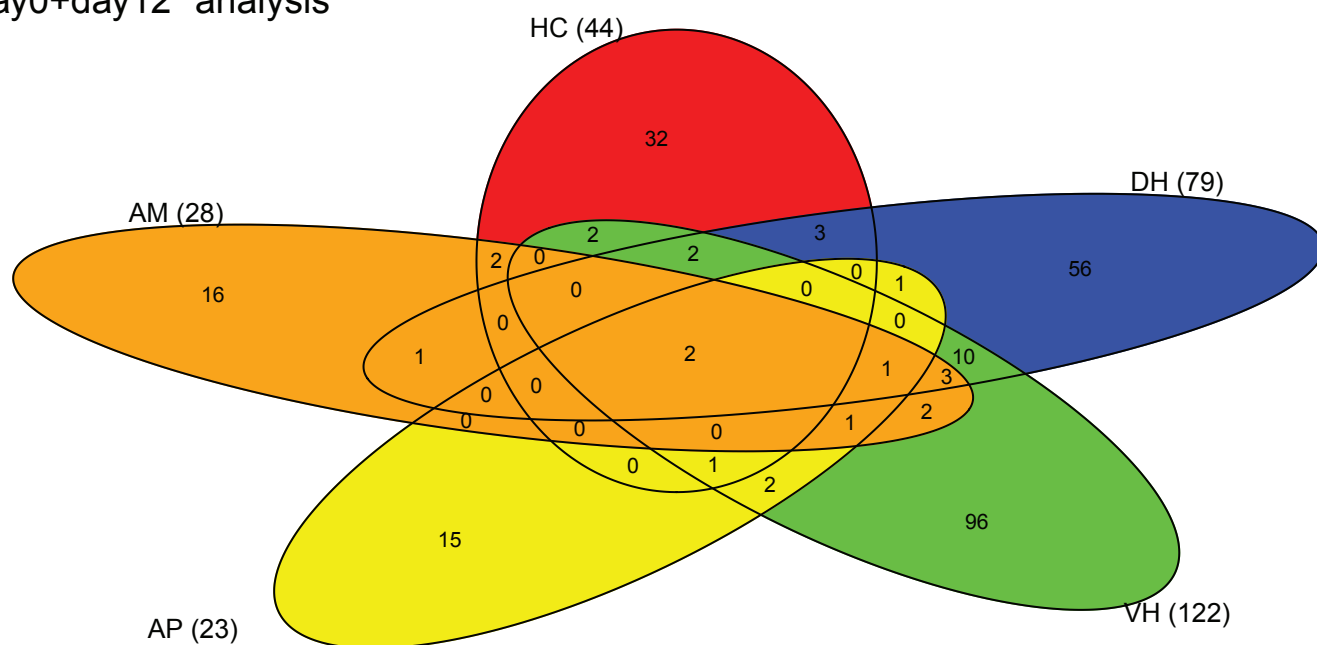

Supplement: Additional file 2 — Venn diagrams showing the number of overlapping probes between the different brain areas per analysis. The figures in brackets represent the total number of estrous behavior associated probes found in each brain area per analysis. Here, AM - Amygdala; HC - Hippocampus; DH - Dorsal Hypothalamus; VH - Ventral Hypothalamus; AP - Anterior Pituitary. [file 1471-2164-12-200-S2.PDF]
