# Supplementary material for: Gene expression patterns in four brain areas associate with quantitative measure of estrous behavior in dairy cows
Source: BMC Genomics. 2011 Apr 19;12:200. doi: 10.1186/1471-2164-12-200 (PMC3110153; doi:10.1186/1471-2164-12-200)
Supplement: Additional file 3 — Significantly enriched Gene Ontology and KEGG pathway terms in the estrous behavior associated gene lists. Table shows all terms with p < 0.10 and includes gene ontology terms in the 3 categories: Biological Process, Molecular function and Cellular component. [file 1471-2164-12-200-S3.PDF]

Significantly enriched Gene Ontology and KEGG pathway terms in the estrous behavior associated gene lists *

Category	Term	Count	%	PValue	Genes	List Total	Pop Hits	Pop Total	Fold Enrichment	Bonferroni	Benjamini	FDR
day0 Amygdala												
BP	GO:0048589~developmental growth	4	5.63	0.01	GINS1,AKT1,PRKCQ,TM4SF4	53	65	8330	9.67	1.00	1.00	11.26
	GO:0007204~elevation of cytosolic calcium ion concentration	4	5.63	0.01	CALCR,MCHR1,AVP,GNA15	53	72	8330	8.73	1.00	0.99	14.65
	GO:0019229~regulation of vasoconstriction	3	4.23	0.01	EDN3,PRKCQ,AVP	53	27	8330	17.46	1.00	0.97	17.34
	GO:0051480~cytosolic calcium ion homeostasis	4	5.63	0.01	CALCR,MCHR1,AVP,GNA15	53	79	8330	7.96	1.00	0.95	18.46
	GO:0051050~positive regulation of transport	5	7.04	0.02	AKT1,EDN3,MCHR1,PRKCQ,AVP	53	157	8330	5.01	1.00	0.95	22.62
	GO:0051240~positive regulation of multicellular organismal process	5	7.04	0.02	AKT1,EDN3,AVP,SPTBN4,TRADD	53	169	8330	4.65	1.00	0.96	27.94
	GO:0008104~protein localization	10	14.08	0.02	AKT1,SNX27,TOM1L2,RAB19,FXC1,SNX7,SPTBN4,MAPK8IP3,SNX3,NUP107	53	667	8330	2.36	1.00	0.93	28.10
	GO:0007186~G-protein coupled receptor protein signaling pathway	7	9.86	0.02	CALCR,AKT1,MCHR1,AVP,GNA15,GPR19,GABRA6	53	361	8330	3.05	1.00	0.94	31.63
	GO:0030182~neuron differentiation	6	8.45	0.03	EDN3,PRKCQ,SPTBN4,MAPK8IP3,NR2E3,ETV4	53	280	8330	3.37	1.00	0.95	37.52
	GO:0006874~cellular calcium ion homeostasis	4	5.63	0.04	CALCR,MCHR1,AVP,GNA15	53	118	8330	5.33	1.00	0.97	44.51
	GO:0007610~behavior	6	8.45	0.04	EDN3,MCHR1,AVP,SPTBN4,CHRNA5,CCL26	53	303	8330	3.11	1.00	0.96	46.87
	GO:0055074~calcium ion homeostasis	4	5.63	0.04	CALCR,MCHR1,AVP,GNA15	53	123	8330	5.11	1.00	0.96	48.05
	GO:0030817~regulation of cAMP biosynthetic process	3	4.23	0.04	CALCR,MCHR1,AVP	53	52	8330	9.07	1.00	0.95	48.41
	GO:0044093~positive regulation of molecular function	7	9.86	0.04	CALCR,AKT1,EDN3,PRKCQ,GNA15,MAPK8IP3,PLCB2	53	416	8330	2.64	1.00	0.94	50.25
	GO:0030814~regulation of cAMP metabolic process	3	4.23	0.04	CALCR,MCHR1,AVP	53	54	8330	8.73	1.00	0.94	50.83
	GO:0006875~cellular metal ion homeostasis	4	5.63	0.04	CALCR,MCHR1,AVP,GNA15	53	127	8330	4.95	1.00	0.92	50.85
	GO:0040007~growth	4	5.63	0.05	GINS1,AKT1,PRKCQ,TM4SF4	53	130	8330	4.84	1.00	0.92	52.93
	GO:0006091~generation of precursor metabolites and energy	5	7.04	0.05	AKT1,MCHR1,AVP,PHKA1,NDUFS1	53	221	8330	3.56	1.00	0.92	53.93
	GO:0055065~metal ion homeostasis	4	5.63	0.05	CALCR,MCHR1,AVP,GNA15	53	135	8330	4.66	1.00	0.92	56.33
	GO:0030802~regulation of cyclic nucleotide biosynthetic process	3	4.23	0.05	CALCR,MCHR1,AVP	53	60	8330	7.86	1.00	0.92	57.76
	GO:0030808~regulation of nucleotide biosynthetic process	3	4.23	0.05	CALCR,MCHR1,AVP	53	60	8330	7.86	1.00	0.92	57.76
	GO:0045927~positive regulation of growth	3	4.23	0.06	AKT1,AVP,SPTBN4	53	61	8330	7.73	1.00	0.91	58.86
	GO:0019932~second-messenger-mediated signaling	4	5.63	0.06	CALCR,EDN3,MCHR1,GNA15	53	139	8330	4.52	1.00	0.90	58.99
	GO:0030799~regulation of cyclic nucleotide metabolic process	3	4.23	0.06	CALCR,MCHR1,AVP	53	63	8330	7.48	1.00	0.91	61.02
	GO:0006140~regulation of nucleotide metabolic process	3	4.23	0.06	CALCR,MCHR1,AVP	53	65	8330	7.25	1.00	0.91	63.11
	GO:0030005~cellular di-, tri-valent inorganic cation homeostasis	4	5.63	0.06	CALCR,MCHR1,AVP,GNA15	53	148	8330	4.25	1.00	0.91	64.72

Category	Term	Count	%	PValue	Genes	List Total	Pop Hits	Pop Total	Fold Enrichment	Bonferroni	Benjamini	FDR
	GO:0015031~protein transport	8	11.27	0.07	AKT1,SNX27,TOM1L2,RAB19,FXC1,SNX7,SNX3,NUP107	53	579	8330	2.17	1.00	0.91	65.91
	GO:0045184~establishment of protein localization	8	11.27	0.07	AKT1,SNX27,TOM1L2,RAB19,FXC1,SNX7,SNX3,NUP107	53	585	8330	2.15	1.00	0.91	67.58
	GO:0009611~response to wounding	6	8.45	0.07	AKT1,PRKCQ,THBD,LTA4H,TM4SF4,CCL26	53	357	8330	2.64	1.00	0.91	68.12
	GO:0035094~response to nicotine	2	2.82	0.07	AVP,CHRNA5	53	12	8330	26.19	1.00	0.90	69.04
	GO:0006873~cellular ion homeostasis	5	7.04	0.07	CALCR,MCHR1,AVP,GNA15,NDUFS1	53	254	8330	3.09	1.00	0.90	69.45
	GO:0051047~positive regulation of secretion	3	4.23	0.07	EDN3,PRKCQ,AVP	53	72	8330	6.55	1.00	0.89	69.89
	GO:0043085~positive regulation of catalytic activity	6	8.45	0.08	CALCR,AKT1,EDN3,GNA15,MAPK8IP3,PLCB2	53	364	8330	2.59	1.00	0.89	70.58
	GO:0055066~di-, tri-valent inorganic cation homeostasis	4	5.63	0.08	CALCR,MCHR1,AVP,GNA15	53	159	8330	3.95	1.00	0.89	71.12
	GO:0055082~cellular chemical homeostasis	5	7.04	0.08	CALCR,MCHR1,AVP,GNA15,NDUFS1	53	258	8330	3.05	1.00	0.88	71.14
	GO:0042310~vasoconstriction	2	2.82	0.08	EDN3,AVP	53	13	8330	24.18	1.00	0.88	71.92
	GO:0031328~positive regulation of cellular biosynthetic process	7	9.86	0.08	AKT1,PRKCQ,AVP,TTF1,NR2E3,MKL1,ETV4	53	484	8330	2.27	1.00	0.87	72.45
	GO:0009891~positive regulation of biosynthetic process	7	9.86	0.08	AKT1,PRKCQ,AVP,TTF1,NR2E3,MKL1,ETV4	53	489	8330	2.25	1.00	0.88	73.87
	GO:0030003~cellular cation homeostasis	4	5.63	0.09	CALCR,MCHR1,AVP,GNA15	53	167	8330	3.76	1.00	0.88	75.32
	GO:0007626~locomotory behavior	4	5.63	0.09	EDN3,AVP,SPTBN4,CCL26	53	169	8330	3.72	1.00	0.88	76.30
	GO:0008633~activation of pro-apoptotic gene products	2	2.82	0.10	AKT1,TRADD	53	16	8330	19.65	1.00	0.90	79.06
	GO:0050801~ion homeostasis	5	7.04	0.10	CALCR,MCHR1,AVP,GNA15,NDUFS1	53	281	8330	2.80	1.00	0.90	79.79
	GO:0009628~response to abiotic stimulus	5	7.04	0.10	AKT1,PRKCQ,AVP,THBD,NR2E3	53	282	8330	2.79	1.00	0.89	80.12
	GO:0005543~phospholipid binding	5	7.04	0.01	AKT1,SNX27,SNX7,SNX3,PLCB2	52	143	7910	5.32	0.92	0.92	15.32
	GO:0035091~phosphoinositide binding	4	5.63	0.02	AKT1,SNX27,SNX7,SNX3	52	84	7910	7.24	0.96	0.80	18.90
	GO:0008289~lipid binding	7	9.86	0.02	AKT1,PRKCQ,SNX27,SNX7,SNX3,NR2E3,PLCB2	52	331	7910	3.22	0.97	0.71	21.16
MF	GO:0030594~neurotransmitter receptor activity	3	4.23	0.04	MCHR1,GABRA6,CHRNA5	52	46	7910	9.92	1.00	0.82	36.01
	GO:0042165~neurotransmitter binding	3	4.23	0.04	MCHR1,GABRA6,CHRNA5	52	52	7910	8.78	1.00	0.82	42.92
	GO:0015276~ligand-gated ion channel activity	3	4.23	0.08	KCNJ15,GABRA6,CHRNA5	52	75	7910	6.08	1.00	0.94	66.46
	GO:0022834~ligand-gated channel activity	3	4.23	0.08	KCNJ15,GABRA6,CHRNA5	52	75	7910	6.08	1.00	0.94	66.46

Category	Term	Count	%	PValue	Genes	List Total	Pop Hits	Pop Total	Fold Enrichment	Bonferroni	Benjamini	FDR
CC	GO:0044459~plasma membrane part	20	28.17	0.00	CALCR,MCHR1,KCNJ15,ATP1B1,GNA15,GPR19,GABRA6,PHKA1,CTLA4,TSPAN15,TSPAN9,PVRL4,PRKCQ,DSG4,THBD,RAB19,CHRNA5,MAPK8IP3,TM4SF4,TES	48	1491	7836	2.19	0.08	0.08	0.65
	GO:0042995~cell projection	10	14.08	0.00	CALCR,AKT1,DNAH11,MCHR1,AVP,GABRA6,SPTBN4,CHRNA5,MAPK8IP3,GAS8	48	514	7836	3.18	0.39	0.22	3.61
	GO:0005887~integral to plasma membrane	12	16.90	0.00	CALCR,KCNJ15,MCHR1,ATP1B1,THBD,GPR19,GABRA6,CHRNA5,CTLA4,TSPAN9,TSPAN15,TM4SF4	48	768	7836	2.55	0.54	0.23	5.68
	GO:0031226~intrinsic to plasma membrane	12	16.90	0.01	CALCR,KCNJ15,MCHR1,ATP1B1,THBD,GPR19,GABRA6,CHRNA5,CTLA4,TSPAN9,TSPAN15,TM4SF4	48	787	7836	2.49	0.61	0.21	6.80
	GO:0005625~soluble fraction	6	8.45	0.01	AKT1,EDN3,PRKCQ,AVP,MAPK8IP3,TEP1	48	230	7836	4.26	0.85	0.32	13.31
	GO:0005886~plasma membrane	22	30.99	0.01	CALCR,MCHR1,KCNJ15,ATP1B1,GNA15,GPR19,GABRA6,PHKA1,CTLA4,TMPRSS7,TSPAN15,TSPAN9,AKT1,PVRL4,PRKCQ,DSG4,THBD,RAB19,CHRNA5,MAPK8IP3,TM4SF4,TES	48	2220	7836	1.62	0.86	0.28	13.60
	GO:0043005~neuron projection	6	8.45	0.02	CALCR,AVP,GABRA6,SPTBN4,CHRNA5,MAPK8IP3	48	256	7836	3.83	0.95	0.34	19.67
	GO:0044463~cell projection part	5	7.04	0.02	CALCR,DNAH11,SPTBN4,MAPK8IP3,GAS8	48	173	7836	4.72	0.96	0.33	21.28
	GO:0030425~dendrite	4	5.63	0.04	AVP,GABRA6,CHRNA5,MAPK8IP3	48	121	7836	5.40	1.00	0.48	35.51
	GO:0043025~cell soma	4	5.63	0.04	CALCR,GABRA6,SPTBN4,CHRNA5	48	126	7836	5.18	1.00	0.48	38.53
	GO:0070161~anchoring junction	4	5.63	0.05	PVRL4,KCNJ15,DSG4,TES	48	136	7836	4.80	1.00	0.51	44.61
	GO:0009434~microtubule-based flagellum	2	2.82	0.07	CALCR,GAS8	48	12	7836	27.21	1.00	0.62	58.28
	GO:0030054~cell junction	6	8.45	0.07	PVRL4,KCNJ15,DSG4,GABRA6,CHRNA5,TES	48	371	7836	2.64	1.00	0.59	58.38
	GO:0043235~receptor complex	3	4.23	0.09	GABRA6,CHRNA5,TRADD	48	85	7836	5.76	1.00	0.67	68.86
	GO:0000267~cell fraction	9	12.68	0.09	AKT1,EDN3,PRKCQ,AVP,MAPK8IP3,TEP1,TSPAN9,TSPAN15,TM4SF4	48	785	7836	1.87	1.00	0.65	69.26
KP	hsa04920:Adipocytokine signaling pathway	3	4.23	0.04	AKT1,PRKCQ,TRADD	18	60	3184	8.84	0.90	0.90	33.01
	hsa04270:Vascular smooth muscle contraction	3	4.23	0.07	PRKCQ,PLCB2,MYL9	18	80	3184	6.63	0.98	0.86	49.42
	hsa04660:T cell receptor signaling pathway	3	4.23	0.08	AKT1,PRKCQ,CTLA4	18	87	3184	6.10	0.99	0.78	54.80

Category	Term	Count	%	PValue	Genes	List Total	Pop Hits	Pop Total	Fold Enrichment	Bonferroni	Benjamini	FDR
	hsa04530:Tight junction	3	4.23	0.08	AKT1,PRKCQ,MYL9	18	90	3184	5.90	0.99	0.70	57.02
day0 Hippocampus												
BP	GO:0046903~secretion	5	20.00	0.00	CGA,LAT,AVP,BAIAP3,OXT	21	207	8330	9.58	0.45	0.45	1.85
	GO:0002118~aggressive behavior	2	8.00	0.01	AVP,OXT	21	3	8330	264.44	0.96	0.80	9.73
	GO:0002125~maternal aggressive behavior	2	8.00	0.01	AVP,OXT	21	3	8330	264.44	0.96	0.80	9.73
	GO:0060180~female mating behavior	2	8.00	0.01	AVP,OXT	21	4	8330	198.33	0.99	0.76	12.75
	GO:0045924~regulation of female receptivity	2	8.00	0.01	AVP,OXT	21	4	8330	198.33	0.99	0.76	12.75
	GO:0007267~cell-cell signaling	5	20.00	0.01	CGA,AVP,BAIAP3,OXT,POMC	21	366	8330	5.42	0.99	0.68	13.48
	GO:0008217~regulation of blood pressure	3	12.00	0.01	AVP,OXT,POMC	21	69	8330	17.25	0.99	0.65	15.34
	GO:0042711~maternal behavior	2	8.00	0.01	AVP,OXT	21	5	8330	158.67	1.00	0.59	15.68
	GO:0060746~parental behavior	2	8.00	0.01	AVP,OXT	21	5	8330	158.67	1.00	0.59	15.68
	GO:0033057~reproductive behavior in a multicellular organism	2	8.00	0.01	AVP,OXT	21	6	8330	132.22	1.00	0.60	18.51
	GO:0042538~hyperosmotic salinity response	2	8.00	0.01	AVP,OXT	21	6	8330	132.22	1.00	0.60	18.51
	GO:0050891~multicellular organismal water homeostasis	2	8.00	0.02	AVP,OXT	21	7	8330	113.33	1.00	0.61	21.24
	GO:0003078~regulation of natriuresis	2	8.00	0.02	AVP,OXT	21	7	8330	113.33	1.00	0.61	21.24
	GO:0007625~grooming behavior	2	8.00	0.02	AVP,OXT	21	7	8330	113.33	1.00	0.61	21.24
	GO:0044062~regulation of excretion	2	8.00	0.02	AVP,OXT	21	8	8330	99.17	1.00	0.62	23.89
	GO:0007617~mating behavior	2	8.00	0.02	AVP,OXT	21	8	8330	99.17	1.00	0.62	23.89
	GO:0007620~copulation	2	8.00	0.02	AVP,OXT	21	9	8330	88.15	1.00	0.62	26.44
	GO:0050878~regulation of body fluid levels	3	12.00	0.02	AVP,OXT,GNA12	21	97	8330	12.27	1.00	0.60	27.33
	GO:0035176~social behavior	2	8.00	0.02	AVP,OXT	21	10	8330	79.33	1.00	0.59	28.91
	GO:0019098~reproductive behavior	2	8.00	0.02	AVP,OXT	21	10	8330	79.33	1.00	0.59	28.91
	GO:0009651~response to salt stress	2	8.00	0.03	AVP,OXT	21	11	8330	72.12	1.00	0.60	31.30
	GO:0030104~water homeostasis	2	8.00	0.03	AVP,OXT	21	11	8330	72.12	1.00	0.60	31.30
	GO:0006972~hyperosmotic response	2	8.00	0.03	AVP,OXT	21	12	8330	66.11	1.00	0.60	33.60
	GO:0009628~response to abiotic stimulus	4	16.00	0.03	AVP,OXT,TRPV3,SMC1A	21	282	8330	5.63	1.00	0.58	33.71
	GO:0007618~mating	2	8.00	0.03	AVP,OXT	21	14	8330	56.67	1.00	0.61	37.99
	GO:0045777~positive regulation of blood pressure	2	8.00	0.04	AVP,OXT	21	16	8330	49.58	1.00	0.64	42.08
	GO:0008015~blood circulation	3	12.00	0.04	AVP,OXT,POMC	21	132	8330	9.02	1.00	0.63	43.38
	GO:0003013~circulatory system process	3	12.00	0.04	AVP,OXT,POMC	21	132	8330	9.02	1.00	0.63	43.38
	GO:0051705~behavioral interaction between organisms	2	8.00	0.04	AVP,OXT	21	18	8330	44.07	1.00	0.64	45.91
	GO:0032940~secretion by cell	3	12.00	0.05	CGA,LAT,BAIAP3	21	145	8330	8.21	1.00	0.66	49.18

Category	Term	Count	%	PValue	Genes	List Total	Pop Hits	Pop Total	Fold Enrichment	Bonferroni	Benjamini	FDR
	GO:0007186~G-protein coupled receptor protein signaling pathway	4	16.00	0.05	CGA,AVP,GNA12,POMC	21	361	8330	4.40	1.00	0.69	54.00
	GO:0007166~cell surface receptor linked signal transduction	6	24.00	0.06	CGA,LAT,AVP,NKD1,GNA12,POMC	21	895	8330	2.66	1.00	0.69	55.77
	GO:0006970~response to osmotic stress	2	8.00	0.06	AVP,OXT	21	27	8330	29.38	1.00	0.72	60.24
	GO:0032846~positive regulation of homeostatic process	2	8.00	0.08	AVP,OXT	21	33	8330	24.04	1.00	0.77	67.62
	GO:0043279~response to alkaloid	2	8.00	0.09	AVP,OXT	21	38	8330	20.88	1.00	0.81	72.71
MF	GO:0005179~hormone activity	5	20.00	0.00	CGA,TTR,AVP,OXT,POMC	21	51	7910	36.93	0.00	0.00	0.01
	GO:0031895~V1B vasopressin receptor binding	2	8.00	0.01	AVP,OXT	21	2	7910	376.67	0.32	0.18	5.20
	GO:0031855~oxytocin receptor binding	2	8.00	0.01	AVP,OXT	21	2	7910	376.67	0.32	0.18	5.20
	GO:0031893~vasopressin receptor binding	2	8.00	0.01	AVP,OXT	21	3	7910	251.11	0.44	0.18	7.70
	GO:0031894~V1A vasopressin receptor binding	2	8.00	0.01	AVP,OXT	21	3	7910	251.11	0.44	0.18	7.70
	GO:0005184~neuropeptide hormone activity	2	8.00	0.03	AVP,OXT	21	10	7910	75.33	0.86	0.39	23.45
CC	GO:0005625~soluble fraction	4	16.00	0.01	CGA,AVP,GPX3,POMC	18	230	7836	7.57	0.58	0.58	12.15
	GO:0030141~secretory granule	3	12.00	0.03	AVP,OXT,POMC	18	123	7836	10.62	0.86	0.63	25.73
	GO:0005615~extracellular space	4	16.00	0.06	TTR,AVP,OXT,GPX3	18	408	7836	4.27	0.98	0.72	44.32
	GO:0005576~extracellular region	6	24.00	0.07	CGA,TTR,AVP,OXT,GPX3,POMC	18	1081	7836	2.42	0.99	0.73	54.51
	GO:0000267~cell fraction	5	20.00	0.08	CGA,AVP,GPX3,TCL1A,POMC	18	785	7836	2.77	1.00	0.69	58.93
	GO:0000777~condensed chromosome kinetochore	2	8.00	0.09	NUP107,SMC1A	18	44	7836	19.79	1.00	0.66	62.69
KP	NIL											
day0 Dorsal Hypothalamus												
	GO:0051591~response to cAMP	3	14.29	0.00	THBD,OXT,PEBP1	17	31	8330	47.42	0.57	0.57	2.25
	GO:0044057~regulation of system process	4	19.05	0.01	SLC1A3,OXT,ATP2A1,PEBP1	17	210	8330	9.33	0.98	0.85	9.65
	GO:0007565~female pregnancy	3	14.29	0.01	THBD,OXT,GNAS	17	77	8330	19.09	0.99	0.82	12.74
	GO:0050804~regulation of synaptic transmission	3	14.29	0.01	SLC1A3,OXT,PEBP1	17	91	8330	16.15	1.00	0.83	17.15
	GO:0016055~Wnt receptor signaling pathway	3	14.29	0.01	NKD1,DKK1,MARK4	17	96	8330	15.31	1.00	0.79	18.82
	GO:0051969~regulation of transmission of nerve impulse	3	14.29	0.02	SLC1A3,OXT,PEBP1	17	100	8330	14.70	1.00	0.76	20.18
	GO:0009628~response to abiotic stimulus	4	19.05	0.02	SLC1A3,THBD,OXT,PEBP1	17	282	8330	6.95	1.00	0.70	20.36
	GO:0031644~regulation of neurological system process	3	14.29	0.02	SLC1A3,OXT,PEBP1	17	105	8330	14.00	1.00	0.69	21.92
	GO:0051602~response to electrical stimulus	2	9.52	0.02	OXT,PEBP1	17	9	8330	108.89	1.00	0.65	22.31
	GO:0014823~response to activity	2	9.52	0.02	OXT,PEBP1	17	10	8330	98.00	1.00	0.65	24.45
	GO:0042755~eating behavior	2	9.52	0.02	OXT,PEBP1	17	13	8330	75.38	1.00	0.71	30.56
	GO:0045933~positive regulation of muscle contraction	2	9.52	0.03	OXT,ATP2A1	17	15	8330	65.33	1.00	0.73	34.35
	GO:0050806~positive regulation of synaptic transmission	2	9.52	0.04	SLC1A3,OXT	17	19	8330	51.58	1.00	0.78	41.32

Category	Term	Count	%	PValue	Genes	List Total	Pop Hits	Pop Total	Fold Enrichment	Bonferroni	Benjamini	FDR
BP	GO:0051971~positive regulation of transmission of nerve impulse	2	9.52	0.04	SLC1A3,OXT	17	20	8330	49.00	1.00	0.78	42.95
	GO:0009968~negative regulation of signal transduction	3	14.29	0.04	DKK1,PEBP1,CYP26A1	17	167	8330	8.80	1.00	0.77	44.75
	GO:0051240~positive regulation of multicellular organismal process	3	14.29	0.04	SLC1A3,OXT,ATP2A1	17	169	8330	8.70	1.00	0.76	45.48
	GO:0031646~positive regulation of neurological system process	2	9.52	0.04	SLC1A3,OXT	17	22	8330	44.55	1.00	0.74	46.07
	GO:0042493~response to drug	3	14.29	0.04	SLC1A3,PEBP1,GNAS	17	171	8330	8.60	1.00	0.72	46.20
	GO:0007268~synaptic transmission	3	14.29	0.05	SLC1A3,OXT,PEBP1	17	183	8330	8.03	1.00	0.75	50.49
	GO:0010648~negative regulation of cell communication	3	14.29	0.05	DKK1,PEBP1,CYP26A1	17	185	8330	7.95	1.00	0.74	51.19
	GO:0019226~transmission of nerve impulse	3	14.29	0.06	SLC1A3,OXT,PEBP1	17	219	8330	6.71	1.00	0.82	62.36
	GO:0010647~positive regulation of cell communication	3	14.29	0.07	SLC1A3,OXT,PEBP1	17	225	8330	6.53	1.00	0.83	64.17
	GO:0050877~neurological system process	4	19.05	0.07	SLC1A3,OXT,PEBP1,GNAS	17	508	8330	3.86	1.00	0.82	65.17
	GO:0007631~feeding behavior	2	9.52	0.07	OXT,PEBP1	17	38	8330	25.79	1.00	0.81	65.61
	GO:0044271~nitrogen compound biosynthetic process	3	14.29	0.07	MOCOS,SLC1A3,ATP2A1	17	233	8330	6.31	1.00	0.81	66.50
	GO:0001505~regulation of neurotransmitter levels	2	9.52	0.08	SLC1A3,PEBP1	17	45	8330	21.78	1.00	0.84	71.77
	GO:0010033~response to organic substance	4	19.05	0.09	THBD,OXT,PEBP1,GNAS	17	562	8330	3.49	1.00	0.85	74.20
	GO:0010243~response to organic nitrogen	2	9.52	0.09	OXT,PEBP1	17	49	8330	20.00	1.00	0.84	74.78
	GO:0006937~regulation of muscle contraction	2	9.52	0.09	OXT,ATP2A1	17	50	8330	19.60	1.00	0.84	75.48
	GO:0008016~regulation of heart contraction	2	9.52	0.09	OXT,PEBP1	17	50	8330	19.60	1.00	0.84	75.48
MF	GO:0005179~hormone activity	2	9.52	0.09	TTR,OXT	16	51	7910	19.39	1.00	1.00	67.47
	GO:0005509~calcium ion binding	4	19.05	0.10	NKD1,THBD,ATP2A1,CACNA1D	16	598	7910	3.31	1.00	1.00	70.06
CC	GO:0005624~membrane fraction	6	28.57	0.00	SLC1A3,ATP2A1,TCL1A,PEBP1,CYP26A1, GNAS	16	587	7836	5.01	0.33	0.33	4.10
	GO:0005626~insoluble fraction	6	28.57	0.00	SLC1A3,ATP2A1,TCL1A,PEBP1,CYP26A1, GNAS	16	612	7836	4.80	0.39	0.22	4.90
	GO:0005576~extracellular region	7	33.33	0.01	TTR,SLC1A3,DKK1,THBD,OXT,PEBP1,GN AS	16	1081	7836	3.17	0.71	0.34	11.95
	GO:0043005~neuron projection	4	19.05	0.01	SLC1A3,OXT,PEBP1,MARK4	16	256	7836	7.65	0.73	0.28	12.47
	GO:0000267~cell fraction	6	28.57	0.01	SLC1A3,ATP2A1,TCL1A,PEBP1,CYP26A1, GNAS	16	785	7836	3.74	0.76	0.25	13.47
	GO:0042995~cell projection	5	23.81	0.01	SLC1A3,OXT,PEBP1,GNAS,MARK4	16	514	7836	4.76	0.79	0.23	14.70
	GO:0044421~extracellular region part	5	23.81	0.02	TTR,SLC1A3,THBD,OXT,PEBP1	16	601	7836	4.07	0.93	0.31	23.70
	GO:0034704~calcium channel complex	2	9.52	0.04	ATP2A1,CACNA1D	16	20	7836	48.98	0.99	0.41	35.18
	GO:0005792~microsome	3	14.29	0.04	ATP2A1,TCL1A,CYP26A1	16	165	7836	8.90	0.99	0.38	35.95
	GO:0005615~extracellular space	4	19.05	0.04	TTR,THBD,OXT,PEBP1	16	408	7836	4.80	0.99	0.36	36.92
	GO:0042598~vesicular fraction	3	14.29	0.04	ATP2A1,TCL1A,CYP26A1	16	171	7836	8.59	0.99	0.34	37.88

Category	Term	Count	%	PValue	Genes	List Total	Pop Hits	Pop Total	Fold Enrichment	Bonferroni	Benjamini	FDR
	GO:0044463~cell projection part	3	14.29	0.04	SLC1A3,OXT,PEBP1	16	173	7836	8.49	0.99	0.33	38.52
	GO:0043679~nerve terminal	2	9.52	0.05	OXT,PEBP1	16	27	7836	36.28	1.00	0.35	44.32
	GO:0031090~organelle membrane	5	23.81	0.06	SLC1A3,ATP2A1,PEBP1,CYP26A1,GNAS	16	785	7836	3.12	1.00	0.36	47.66
	GO:0033267~axon part	2	9.52	0.07	OXT,PEBP1	16	39	7836	25.12	1.00	0.42	57.10
KP	hsa04020:Calcium signaling pathway	3	14.29	0.03	ATP2A1,GNAS,CACNA1D	9	109	3184	9.74	0.48	0.48	20.43
day0 Ventral Hypothalamus												
BP	GO:0006817~phosphate transport	2	7.69	0.02	SLC17A7,MARCO	19	10	8330	87.68	1.00	1.00	24.63
	GO:0045596~negative regulation of cell differentiation	3	11.54	0.04	CTLA4,TCL1A,LIG4	19	147	8330	8.95	1.00	0.99	40.80
	GO:0019827~stem cell maintenance	2	7.69	0.04	TCL1A,LIG4	19	19	8330	46.15	1.00	0.97	41.58
	GO:0048864~stem cell development	2	7.69	0.04	TCL1A,LIG4	19	20	8330	43.84	1.00	0.94	43.21
	GO:0048863~stem cell differentiation	2	7.69	0.05	TCL1A,LIG4	19	23	8330	38.12	1.00	0.92	47.84
	GO:0050877~neurological system process	4	15.38	0.09	SLC17A7,DLGAP1,NEUROD2,SIX6	19	508	8330	3.45	1.00	0.99	72.08
MF	NIL											
CC	GO:0045202~synapse	4	15.38	0.01	SLC17A7,DLGAP1,SPARCL1,LRRK2	15	250	7836	8.36	0.54	0.54	9.29
	GO:0030136~clathrin-coated vesicle	3	11.54	0.01	SLC17A7,CTLA4,LRRK2	15	101	7836	15.52	0.69	0.44	13.66
	GO:0030135~coated vesicle	3	11.54	0.02	SLC17A7,CTLA4,LRRK2	15	126	7836	12.44	0.83	0.45	20.08
	GO:0044456~synapse part	3	11.54	0.04	SLC17A7,DLGAP1,LRRK2	15	169	7836	9.27	0.96	0.54	32.28
	GO:0005886~plasma membrane	8	30.77	0.07	SLC17A7,MARCO,DLGAP1,NKD1,CTLA4,SNX1,LIG4,LRRK2	15	2220	7836	1.88	1.00	0.72	54.81
	GO:0008021~synaptic vesicle	2	7.69	0.09	SLC17A7,LRRK2	15	52	7836	20.09	1.00	0.74	63.43
KP	hsa05320:Autoimmune thyroid disease	2	7.69	0.03	CGA,CTLA4	6	19	3184	55.86	0.24	0.24	15.83
day0 Anterior Pituitary												
	GO:0007610~behavior	7	16.28	0.00	PLP2,SLC1A3,MCOLN3,ENPP2,ATP1A3,NEUROD2,ATP1A2	34	303	8330	5.66	0.43	0.43	1.53
	GO:0050877~neurological system process	8	18.60	0.00	PLAT,SLC1A3,MCOLN3,SYN2,ATP1A3,NEUROD2,ATP1A2,CLDN11	34	508	8330	3.86	0.82	0.57	4.56
	GO:0007626~locomotory behavior	5	11.63	0.00	PLP2,MCOLN3,ENPP2,ATP1A3,ATP1A2	34	169	8330	7.25	0.89	0.53	5.97
	GO:0019226~transmission of nerve impulse	5	11.63	0.01	PLAT,SLC1A3,SYN2,ATP1A2,CLDN11	34	219	8330	5.59	1.00	0.75	14.16
	GO:0007612~learning	3	6.98	0.01	ATP1A3,NEUROD2,ATP1A2	34	43	8330	17.09	1.00	0.73	16.60
	GO:0001505~regulation of neurotransmitter levels	3	6.98	0.01	SLC1A3,SYN2,ATP1A2	34	45	8330	16.33	1.00	0.70	17.97
	GO:0006811~ion transport	7	16.28	0.01	PLP2,MLC1,SLC1A3,MCOLN3,ATP1A3,ATP1A2,ANO8	34	514	8330	3.34	1.00	0.66	18.82

Category	Term	Count	%	PValue	Genes	List Total	Pop Hits	Pop Total	Fold Enrichment	Bonferroni	Benjamini	FDR
BP	GO:0006836~neurotransmitter transport	3	6.98	0.02	SLC1A3,SYN2,ATP1A2	34	49	8330	15.00	1.00	0.66	20.81
	GO:0030182~neuron differentiation	5	11.63	0.02	SLC1A3,MCOLN3,OPCML,STMN2,NEUROD2	34	280	8330	4.38	1.00	0.76	29.47
	GO:0009628~response to abiotic stimulus	5	11.63	0.02	SLC1A3,ATP1A3,CA2,ATP1A2,THBS1	34	282	8330	4.34	1.00	0.73	30.05
	GO:0001504~neurotransmitter uptake	2	4.65	0.03	SLC1A3,ATP1A2	34	8	8330	61.25	1.00	0.78	36.96
	GO:0007268~synaptic transmission	4	9.30	0.04	PLAT,SLC1A3,SYN2,ATP1A2	34	183	8330	5.36	1.00	0.79	40.50
	GO:0050890~cognition	5	11.63	0.04	SLC1A3,MCOLN3,ATP1A3,NEUROD2,ATP1A2	34	318	8330	3.85	1.00	0.77	40.99
	GO:0007611~learning or memory	3	6.98	0.04	ATP1A3,NEUROD2,ATP1A2	34	79	8330	9.30	1.00	0.78	43.71
	GO:0009416~response to light stimulus	3	6.98	0.06	SLC1A3,ATP1A3,ATP1A2	34	98	8330	7.50	1.00	0.88	57.45
	GO:0008542~visual learning	2	4.65	0.06	ATP1A3,ATP1A2	34	16	8330	30.63	1.00	0.88	60.27
	GO:0007632~visual behavior	2	4.65	0.08	ATP1A3,ATP1A2	34	20	8330	24.50	1.00	0.92	68.47
	GO:0010811~positive regulation of cell-substrate adhesion	2	4.65	0.09	CCDC80,THBS1	34	25	8330	19.60	1.00	0.95	76.38
MF	GO:0001968~fibronectin binding	2	4.65	0.02	CCDC80,THBS1	31	5	7910	102.06	0.95	0.95	20.52
	GO:0005391~sodium:potassium-exchanging ATPase activity	2	4.65	0.03	ATP1A3,ATP1A2	31	8	7910	63.79	0.99	0.91	30.76
	GO:0030247~polysaccharide binding	3	6.98	0.06	ENPP2,CCDC80,THBS1	31	110	7910	6.96	1.00	0.97	55.49
	GO:0001871~pattern binding	3	6.98	0.06	ENPP2,CCDC80,THBS1	31	110	7910	6.96	1.00	0.97	55.49
CC	GO:0005576~extracellular region	11	25.58	0.01	PLAT,SLC1A3,ENPP2,RNASE4,CCDC80,FAM5C,CA2,THBS1,A2ML1,FAM131A,TAC3	34	1081	7836	2.35	0.71	0.71	11.76
	GO:0000267~cell fraction	9	20.93	0.01	PLP2,MLC1,SLC1A3,MOBP,STMN2,TCL1A,SYTL2,ATP1A2,TAC3	34	785	7836	2.64	0.80	0.55	14.82
	GO:0005890~sodium:potassium-exchanging ATPase complex	2	4.65	0.02	ATP1A3,ATP1A2	34	5	7836	92.19	0.91	0.55	21.28
	GO:0043005~neuron projection	5	11.63	0.02	NGEF,SLC1A3,STMN2,CA2,ATP1A2	34	256	7836	4.50	0.91	0.46	21.96
	GO:0005886~plasma membrane	16	37.21	0.03	PLP2,NGEF,PLXNC1,OPCML,ENPP2,STMN2,ATP1A3,CLDN11,ATP1A2,SLC1A3,HEPACAM,MCOLN3,SYN2,SYTL2,CA2,THBS1	34	2220	7836	1.66	0.95	0.45	26.29
	GO:0005624~membrane fraction	7	16.28	0.03	PLP2,MLC1,SLC1A3,STMN2,TCL1A,SYTL2,ATP1A2	34	587	7836	2.75	0.98	0.47	32.10
	GO:0044459~plasma membrane part	12	27.91	0.04	SLC1A3,HEPACAM,OPCML,ENPP2,STMN2,SYN2,ATP1A3,SYTL2,CA2,ATP1A2,CLDN11,THBS1	34	1491	7836	1.85	0.98	0.45	34.42
	GO:0044421~extracellular region part	7	16.28	0.04	PLAT,SLC1A3,CCDC80,CA2,THBS1,A2ML1,TAC3	34	601	7836	2.68	0.99	0.41	34.85

Category	Term	Count	%	PValue	Genes	List Total	Pop Hits	Pop Total	Fold Enrichment	Bonferroni	Benjamini	FDR
	GO:0005626~insoluble fraction	7	16.28	0.04	PLP2,MLC1,SLC1A3,STMN2,TCL1A,SYTL2,ATP1A2	34	612	7836	2.64	0.99	0.40	37.07
	GO:0045202~synapse	4	9.30	0.09	PLAT,SLC1A3,SYN2,ATP1A2	34	250	7836	3.69	1.00	0.64	64.30
	GO:0005615~extracellular space	5	11.63	0.09	PLAT,CA2,THBS1,A2ML1,TAC3	34	408	7836	2.82	1.00	0.62	65.77
KP	NIL											
day12 Amygdala												
BP	GO:0007166~cell surface receptor linked signal transduction	6	22.22	0.03	CGA,LAT,GNA15,NKD1,SMAD1,GLI2	18	895	8330	3.10	1.00	1.00	33.93
	GO:0001649~osteoblast differentiation	2	7.41	0.06	SMAD1,GLI2	18	28	8330	33.06	1.00	1.00	55.17
	GO:0030902~hindbrain development	2	7.41	0.07	SMAD1,GLI2	18	34	8330	27.22	1.00	1.00	62.26
	GO:0035270~endocrine system development	2	7.41	0.08	CGA,GLI2	18	41	8330	22.57	1.00	1.00	69.14
	GO:0042592~homeostatic process	4	14.81	0.09	GNA15,TEP1,SMAD1,NDUFS1	18	519	8330	3.57	1.00	1.00	71.29
MF	NIL											
CC	NIL											
KP	NIL											
day12 Hippocampus												
BP	NIL											
MF	GO:0008047~enzyme activator activity	4	11.76	0.03	GMFB,WDR67,BNIP2,AHSA2	24	245	7910	5.38	0.99	0.99	32.38
CC	GO:0044459~plasma membrane part	8	23.53	0.04	LAT,ACHE,FLT1,NCF2,SLC16A7,PHKB,SLC13A4,PTGFR	19	1491	7836	2.21	0.93	0.93	34.02
	GO:0005887~integral to plasma membrane	5	14.71	0.09	FLT1,NCF2,SLC16A7,SLC13A4,PTGFR	19	768	7836	2.69	1.00	0.96	62.68
	GO:0031226~intrinsic to plasma membrane	5	14.71	0.10	FLT1,NCF2,SLC16A7,SLC13A4,PTGFR	19	787	7836	2.62	1.00	0.89	65.39
KP	NIL											
day12 Dorsal Hypothalamus												
	GO:0032846~positive regulation of homeostatic process	3	10.71	0.00	AVP,CCK,OXT	21	33	8330	36.06	0.70	0.70	3.83
	GO:0002118~aggressive behavior	2	7.14	0.01	AVP,OXT	21	3	8330	264.44	0.96	0.79	9.68
	GO:0002125~maternal aggressive behavior	2	7.14	0.01	AVP,OXT	21	3	8330	264.44	0.96	0.79	9.68
	GO:0060180~female mating behavior	2	7.14	0.01	AVP,OXT	21	4	8330	198.33	0.98	0.75	12.69
	GO:0045924~regulation of female receptivity	2	7.14	0.01	AVP,OXT	21	4	8330	198.33	0.98	0.75	12.69
	GO:0060746~parental behavior	2	7.14	0.01	AVP,OXT	21	5	8330	158.67	0.99	0.73	15.60
	GO:0042711~maternal behavior	2	7.14	0.01	AVP,OXT	21	5	8330	158.67	0.99	0.73	15.60
	GO:0046903~secretion	4	14.29	0.01	CGA,LAT,AVP,OXT	21	207	8330	7.67	1.00	0.67	16.41
	GO:0042538~hyperosmotic salinity response	2	7.14	0.01	AVP,OXT	21	6	8330	132.22	1.00	0.65	18.42

Category	Term	Count	%	PValue	Genes	List Total	Pop Hits	Pop Total	Fold Enrichment	Bonferroni	Benjamini	FDR
BP	GO:0033057~reproductive behavior in a multicellular organism	2	7.14	0.01	AVP,OXT	21	6	8330	132.22	1.00	0.65	18.42
	GO:0007166~cell surface receptor linked signal transduction	7	25.00	0.02	CGA,LAT,AVP,NKD1,CCK,CHRM1,MARK4	21	895	8330	3.10	1.00	0.62	19.92
	GO:0003078~regulation of natriuresis	2	7.14	0.02	AVP,OXT	21	7	8330	113.33	1.00	0.60	21.14
	GO:0007625~grooming behavior	2	7.14	0.02	AVP,OXT	21	7	8330	113.33	1.00	0.60	21.14
	GO:0051930~regulation of sensory perception of pain	2	7.14	0.02	CCK,OXT	21	7	8330	113.33	1.00	0.60	21.14
	GO:0051931~regulation of sensory perception	2	7.14	0.02	CCK,OXT	21	7	8330	113.33	1.00	0.60	21.14
	GO:0050891~multicellular organismal water homeostasis	2	7.14	0.02	AVP,OXT	21	7	8330	113.33	1.00	0.60	21.14
	GO:0032844~regulation of homeostatic process	3	10.71	0.02	AVP,CCK,OXT	21	85	8330	14.00	1.00	0.57	21.89
	GO:0007617~mating behavior	2	7.14	0.02	AVP,OXT	21	8	8330	99.17	1.00	0.57	23.77
	GO:0044062~regulation of excretion	2	7.14	0.02	AVP,OXT	21	8	8330	99.17	1.00	0.57	23.77
	GO:0007620~copulation	2	7.14	0.02	AVP,OXT	21	9	8330	88.15	1.00	0.57	26.32
	GO:0019098~reproductive behavior	2	7.14	0.02	AVP,OXT	21	10	8330	79.33	1.00	0.58	28.78
	GO:0035176~social behavior	2	7.14	0.02	AVP,OXT	21	10	8330	79.33	1.00	0.58	28.78
	GO:0031644~regulation of neurological system process	3	10.71	0.03	AVP,CCK,OXT	21	105	8330	11.33	1.00	0.58	30.84
	GO:0009651~response to salt stress	2	7.14	0.03	AVP,OXT	21	11	8330	72.12	1.00	0.56	31.15
	GO:0030104~water homeostasis	2	7.14	0.03	AVP,OXT	21	11	8330	72.12	1.00	0.56	31.15
	GO:0006972~hyperosmotic response	2	7.14	0.03	AVP,OXT	21	12	8330	66.11	1.00	0.57	33.45
	GO:0042755~eating behavior	2	7.14	0.03	CCK,OXT	21	13	8330	61.03	1.00	0.57	35.67
	GO:0007618~mating	2	7.14	0.03	AVP,OXT	21	14	8330	56.67	1.00	0.58	37.82
	GO:0045777~positive regulation of blood pressure	2	7.14	0.04	AVP,OXT	21	16	8330	49.58	1.00	0.60	41.91
	GO:0051705~behavioral interaction between organisms	2	7.14	0.04	AVP,OXT	21	18	8330	44.07	1.00	0.63	45.72
	GO:0007186~G-protein coupled receptor protein signaling pathway	4	14.29	0.05	CGA,AVP,CCK,CHRM1	21	361	8330	4.40	1.00	0.69	53.80
	GO:0007267~cell-cell signaling	4	14.29	0.06	CGA,AVP,OXT,CHRM1	21	366	8330	4.34	1.00	0.69	55.04
	GO:0006970~response to osmotic stress	2	7.14	0.06	AVP,OXT	21	27	8330	29.38	1.00	0.72	60.03
	GO:0007205~activation of protein kinase C activity by G-protein coupled receptor protein signaling pathway	2	7.14	0.07	CCK,CHRM1	21	28	8330	28.33	1.00	0.72	61.37
	GO:0043279~response to alkaloid	2	7.14	0.09	AVP,OXT	21	38	8330	20.88	1.00	0.81	72.52
	GO:0007631~feeding behavior	2	7.14	0.09	CCK,OXT	21	38	8330	20.88	1.00	0.81	72.52
	GO:0044057~regulation of system process	3	10.71	0.09	AVP,CCK,OXT	21	210	8330	5.67	1.00	0.80	73.29
MF	GO:0005179~hormone activity	5	17.86	0.00	CGA,TTR,AVP,CCK,OXT	23	51	7910	33.72	0.00	0.00	0.01
	GO:0005184~neuropeptide hormone activity	3	10.71	0.00	AVP,CCK,OXT	23	10	7910	103.17	0.03	0.02	0.36
	GO:0031855~oxytocin receptor binding	2	7.14	0.01	AVP,OXT	23	2	7910	343.91	0.42	0.17	5.98

Category	Term	Count	%	PValue	Genes	List Total	Pop Hits	Pop Total	Fold Enrichment	Bonferroni	Benjamini	FDR
M	GO:0031895~V1B vasopressin receptor binding	2	7.14	0.01	AVP,OXT	23	2	7910	343.91	0.42	0.17	5.98
	GO:0031894~V1A vasopressin receptor binding	2	7.14	0.01	AVP,OXT	23	3	7910	229.28	0.56	0.19	8.84
	GO:0031893~vasopressin receptor binding	2	7.14	0.01	AVP,OXT	23	3	7910	229.28	0.56	0.19	8.84
CC	GO:0043679~nerve terminal	3	10.71	0.00	CCK,OXT,CHRM1	19	27	7836	45.82	0.10	0.10	1.71
	GO:0043005~neuron projection	5	17.86	0.00	AVP,CCK,OXT,CHRM1,MARK4	19	256	7836	8.06	0.14	0.07	2.39
	GO:0033267~axon part	3	10.71	0.00	CCK,OXT,CHRM1	19	39	7836	31.72	0.20	0.07	3.52
	GO:0042995~cell projection	5	17.86	0.03	AVP,CCK,OXT,CHRM1,MARK4	19	514	7836	4.01	0.83	0.36	24.19
	GO:0030424~axon	3	10.71	0.03	CCK,OXT,CHRM1	19	117	7836	10.57	0.85	0.32	25.86
	GO:0030425~dendrite	3	10.71	0.03	AVP,CCK,CHRM1	19	121	7836	10.23	0.87	0.29	27.30
	GO:0043195~terminal button	2	7.14	0.04	CCK,OXT	19	18	7836	45.82	0.93	0.32	34.45
	GO:0044463~cell projection part	3	10.71	0.06	CCK,OXT,CHRM1	19	173	7836	7.15	0.98	0.39	46.10
	GO:0005615~extracellular space	4	14.29	0.06	TTR,AVP,CCK,OXT	19	408	7836	4.04	0.99	0.38	49.00
KP	NIL											
day12 Ventral Hypothalamus												
BP	GO:0019932~second-messenger-mediated signaling	4	6.78	0.03	LAT,GNA15,PIK3C2G,HTR2A	39	139	8330	6.15	1.00	1.00	30.76
	GO:0032940~secretion by cell	4	6.78	0.03	CGA,LAT,UNC13C,MYH10	39	145	8330	5.89	1.00	1.00	33.67
	GO:0048015~phosphoinositide-mediated signaling	3	5.08	0.03	GNA15,PIK3C2G,HTR2A	39	60	8330	10.68	1.00	1.00	36.03
	GO:0007213~muscarinic acetylcholine receptor signaling pathway	2	3.39	0.03	GNA15,CHRM3	39	7	8330	61.03	1.00	0.98	37.10
	GO:0007166~cell surface receptor linked signal transduction	9	15.25	0.05	CGA,LAT,TNFRSF1B,GNA15,CHRM3,LIFR,ATP1A3,ENTPD1,HTR2A	39	895	8330	2.15	1.00	0.99	49.06
	GO:0006887~exocytosis	3	5.08	0.05	LAT,UNC13C,MYH10	39	82	8330	7.81	1.00	0.99	54.94
	GO:0046903~secretion	4	6.78	0.07	CGA,LAT,UNC13C,MYH10	39	207	8330	4.13	1.00	0.99	63.70
	GO:0007186~G-protein coupled receptor protein signaling pathway	5	8.47	0.08	CGA,GNA15,CHRM3,ENTPD1,HTR2A	39	361	8330	2.96	1.00	1.00	70.38
	GO:0017076~purine nucleotide binding	13	22.03	0.05	GNA15,PIK3C2G,ATP1A3,ACADL,ITPKA,DDX6,RAB19,CDC42BPA,TEP1,ENTPD1,AKT3,TOP3B,MYH10	43	1370	7910	1.75	1.00	1.00	45.14
	GO:0000166~nucleotide binding	14	23.73	0.06	GNA15,PIK3C2G,ATP1A3,ACADL,ITPKA,DDX6,SLTM,RAB19,CDC42BPA,TEP1,ENTPD1,AKT3,MYH10,TOP3B	43	1591	7910	1.62	1.00	1.00	55.20
	GO:0008227~amine receptor activity	2	3.39	0.07	CHRM3,HTR2A	43	13	7910	28.30	1.00	0.98	56.86
	GO:0032553~ribonucleotide binding	12	20.34	0.08	GNA15,PIK3C2G,RAB19,CDC42BPA,ATP1A3,TEP1,ENTPD1,ITPKA,AKT3,MYH10,DDX6,TOP3B	43	1311	7910	1.68	1.00	0.96	61.69

Category	Term	Count	%	PValue	Genes	List Total	Pop Hits	Pop Total	Fold Enrichment	Bonferroni	Benjamini	FDR
MF	GO:0032555~purine ribonucleotide binding	12	20.34	0.08	GNA15,PIK3C2G,RAB19,CDC42BPA,ATP1A3,TEP1,ENTPD1,ITPKA,AKT3,MYH10,DDX6,TOP3B	43	1311	7910	1.68	1.00	0.96	61.69
	GO:0030554~adenyl nucleotide binding	11	18.64	0.08	PIK3C2G,CDC42BPA,ATP1A3,TEP1,ENTPD1,ACADL,ITPKA,AKT3,MYH10,DDX6,TOP3B	43	1156	7910	1.75	1.00	0.93	61.96
	GO:0001883~purine nucleoside binding	11	18.64	0.08	PIK3C2G,CDC42BPA,ATP1A3,TEP1,ENTPD1,ACADL,ITPKA,AKT3,MYH10,DDX6,TOP3B	43	1173	7910	1.73	1.00	0.91	64.98
	GO:0001882~nucleoside binding	11	18.64	0.09	PIK3C2G,CDC42BPA,ATP1A3,TEP1,ENTPD1,ACADL,ITPKA,AKT3,MYH10,DDX6,TOP3B	43	1183	7910	1.71	1.00	0.88	66.72
CC	GO:0005886~plasma membrane	20	33.90	0.01	GNA15,ATP1A3,LIFR,CTLA4,SLC29A1,LAT,TNFRSF1B,SYNE2,DES,THBD,CHRM3,RAB19,CDC42BPA,AKAP5,EFR3A,UNC13C,ENTPD1,MYH10,TES,HTR2A	41	2220	7836	1.72	0.63	0.63	8.52
	GO:0044459~plasma membrane part	15	25.42	0.01	GNA15,ATP1A3,LIFR,CTLA4,SLC29A1,LAT,SYNE2,THBD,CHRM3,RAB19,CDC42BPA,ENTPD1,UNC13C,TES,HTR2A	41	1491	7836	1.92	0.81	0.56	13.66
	GO:0044463~cell projection part	4	6.78	0.06	CHRM3,KLC2,MYH10,HTR2A	41	173	7836	4.42	1.00	0.92	49.90
	GO:0030496~midbody	2	3.39	0.06	PSRC1,MYH10	41	13	7836	29.40	1.00	0.89	53.95
	GO:0005887~integral to plasma membrane	8	13.56	0.09	SLC29A1,THBD,CHRM3,LIFR,ATP1A3,CTLA4,ENTPD1,HTR2A	41	768	7836	1.99	1.00	0.92	67.14
KP	hsa04020:Calcium signaling pathway	4	6.78	0.03	GNA15,CHRM3,ITPKA,HTR2A	20	109	3184	5.84	0.76	0.76	22.41
	hsa04660:T cell receptor signaling pathway	3	5.08	0.09	LAT,CTLA4,AKT3	20	87	3184	5.49	1.00	0.94	62.06
day12 Anterior Pituitary												
BP	GO:0030334~regulation of cell migration	4	10.26	0.01	SERPINE2,ENPP2,AGT,CXCL10	32	135	8330	7.71	1.00	1.00	17.69
	GO:0040012~regulation of locomotion	4	10.26	0.02	SERPINE2,ENPP2,AGT,CXCL10	32	148	8330	7.04	1.00	0.99	22.06
	GO:0051270~regulation of cell motion	4	10.26	0.02	SERPINE2,ENPP2,AGT,CXCL10	32	153	8330	6.81	1.00	0.96	23.84
	GO:0007626~locomotory behavior	4	10.26	0.02	PTGDS,CXCL14,ENPP2,CXCL10	32	169	8330	6.16	1.00	0.95	29.84
	GO:0007610~behavior	5	12.82	0.02	PTGDS,CXCL14,ENPP2,AGT,CXCL10	32	303	8330	4.30	1.00	0.92	30.44
	GO:0050877~neurological system process	6	15.38	0.04	SLC17A7,CRB1,LXN,AGT,COL11A1,CRYM	32	508	8330	3.07	1.00	0.96	42.50
	GO:0007186~G-protein coupled receptor protein signaling pathway	5	12.82	0.04	NXPH2,CALY,ENPP2,AGT,CXCL10	32	361	8330	3.61	1.00	0.96	47.16
	GO:0007267~cell-cell signaling	5	12.82	0.05	SLC17A7,CRB1,CXCL14,AGT,CXCL10	32	366	8330	3.56	1.00	0.94	48.64
	GO:0006935~chemotaxis	3	7.69	0.05	CXCL14,ENPP2,CXCL10	32	95	8330	8.22	1.00	0.94	50.97

Category	Term	Count	%	PValue	Genes	List Total	Pop Hits	Pop Total	Fold Enrichment	Bonferroni	Benjamini	FDR
	GO:0042330~taxis	3	7.69	0.05	CXCL14,ENPP2,CXCL10	32	95	8330	8.22	1.00	0.94	50.97
	GO:0001658~branching involved in ureteric bud morphogenesis	2	5.13	0.06	GPC3,AGT	32	17	8330	30.62	1.00	0.96	59.87
	GO:0060675~ureteric bud morphogenesis	2	5.13	0.06	GPC3,AGT	32	17	8330	30.62	1.00	0.96	59.87
	GO:0007600~sensory perception	4	10.26	0.06	CRB1,LXN,COL11A1,CRYM	32	248	8330	4.20	1.00	0.95	61.14
	GO:0048729~tissue morphogenesis	3	7.69	0.07	GPC3,AGT,COL11A1	32	117	8330	6.67	1.00	0.95	64.73
	GO:0001657~ureteric bud development	2	5.13	0.08	GPC3,AGT	32	22	8330	23.66	1.00	0.96	69.33
	GO:0030182~neuron differentiation	4	10.26	0.08	UNC5B,CRB1,PTPRZ1,STMN2	32	280	8330	3.72	1.00	0.96	72.00
	GO:0050906~detection of stimulus involved in sensory perception	2	5.13	0.09	LXN,COL11A1	32	26	8330	20.02	1.00	0.96	75.27
	GO:0001656~metanephros development	2	5.13	0.10	GPC3,AGT	32	28	8330	18.59	1.00	0.96	77.80
MF	GO:0004866~endopeptidase inhibitor activity	3	7.69	0.03	SERPINE2,LXN,AGT	29	71	7910	11.53	0.96	0.96	26.21
	GO:0030414~peptidase inhibitor activity	3	7.69	0.03	SERPINE2,LXN,AGT	29	73	7910	11.21	0.97	0.83	27.41
	GO:0005125~cytokine activity	3	7.69	0.04	CXCL14,IL33,CXCL10	29	91	7910	8.99	1.00	0.83	38.33
	GO:0008009~chemokine activity	2	5.13	0.07	CXCL14,CXCL10	29	21	7910	25.98	1.00	0.91	57.93
	GO:0042379~chemokine receptor binding	2	5.13	0.08	CXCL14,CXCL10	29	23	7910	23.72	1.00	0.88	61.27
CC	GO:0005576~extracellular region	18	46.15	0.00	TF,NXPH2,ENPP2,PTPRZ1,IL33,CXCL10,ANGPTL7,GPC3,SERPINE2,PTGDS,CRB1,CXCL14,CCDC70,AGT,WIF1,COL11A1,CHL1,SPON1	33	1081	7836	3.95	0.00	0.00	0.00
	GO:0044421~extracellular region part	11	28.21	0.00	TF,GPC3,SERPINE2,CXCL14,PTPRZ1,AGT,IL33,COL11A1,CHL1,SPON1,CXCL10	33	601	7836	4.35	0.01	0.00	0.10
	GO:0005578~proteinaceous extracellular matrix	6	15.38	0.00	TF,GPC3,PTPRZ1,COL11A1,CHL1,SPON1	33	223	7836	6.39	0.18	0.07	2.13
	GO:0031012~extracellular matrix	6	15.38	0.00	TF,GPC3,PTPRZ1,COL11A1,CHL1,SPON1	33	244	7836	5.84	0.26	0.07	3.14
	GO:0005615~extracellular space	7	17.95	0.01	TF,GPC3,SERPINE2,CXCL14,AGT,IL33,CXCL10	33	408	7836	4.07	0.44	0.11	5.99
	GO:0048471~perinuclear region of cytoplasm	4	10.26	0.05	TF,MTMR14,PTGDS,STMN2	33	207	7836	4.59	1.00	0.61	44.69
KP	NIL											
day0+day12 Amygdala												
	GO:0043467~regulation of generation of precursor metabolites and energy	2	16.67	0.02	AKT1,ECD	8	24	8330	86.77	1.00	1.00	24.12
	GO:0006073~cellular glucan metabolic process	2	16.67	0.02	AKT1,PHKA1	8	27	8330	77.13	1.00	0.98	26.69
	GO:0044042~glucan metabolic process	2	16.67	0.02	AKT1,PHKA1	8	27	8330	77.13	1.00	0.98	26.69
	GO:0005977~glycogen metabolic process	2	16.67	0.02	AKT1,PHKA1	8	27	8330	77.13	1.00	0.98	26.69

Category	Term	Count	%	PValue	Genes	List Total	Pop Hits	Pop Total	Fold Enrichment	Bonferroni	Benjamini	FDR
BP	GO:0010741~negative regulation of protein kinase cascade	2	16.67	0.02	AKT1,IL1RL1	8	28	8330	74.38	1.00	0.93	27.53
	GO:0010906~regulation of glucose metabolic process	2	16.67	0.02	AKT1,ECD	8	30	8330	69.42	1.00	0.89	29.18
	GO:0006112~energy reserve metabolic process	2	16.67	0.03	AKT1,PHKA1	8	33	8330	63.11	1.00	0.85	31.59
	GO:0010675~regulation of cellular carbohydrate metabolic process	2	16.67	0.03	AKT1,ECD	8	33	8330	63.11	1.00	0.85	31.59
	GO:0006109~regulation of carbohydrate metabolic process	2	16.67	0.03	AKT1,ECD	8	34	8330	61.25	1.00	0.81	32.38
	GO:0044264~cellular polysaccharide metabolic process	2	16.67	0.03	AKT1,PHKA1	8	39	8330	53.40	1.00	0.80	36.17
	GO:0031329~regulation of cellular catabolic process	2	16.67	0.04	AKT1,ECD	8	47	8330	44.31	1.00	0.82	41.80
	GO:0048589~developmental growth	2	16.67	0.05	AKT1,CGA	8	65	8330	32.04	1.00	0.88	52.73
	GO:0048585~negative regulation of response to stimulus	2	16.67	0.06	AKT1,CTLA4	8	71	8330	29.33	1.00	0.88	55.90
	GO:0009894~regulation of catabolic process	2	16.67	0.06	AKT1,ECD	8	71	8330	29.33	1.00	0.88	55.90
	GO:0005976~polysaccharide metabolic process	2	16.67	0.06	AKT1,PHKA1	8	77	8330	27.05	1.00	0.87	58.86
	GO:0032870~cellular response to hormone stimulus	2	16.67	0.08	AKT1,CGA	8	101	8330	20.62	1.00	0.92	68.86
	GO:0015980~energy derivation by oxidation of organic compounds	2	16.67	0.09	AKT1,PHKA1	8	109	8330	19.11	1.00	0.91	71.63
	GO:0006006~glucose metabolic process	2	16.67	0.09	AKT1,PHKA1	8	114	8330	18.27	1.00	0.91	73.23
MF	NIL											
CC	NIL											
KP	hsa05320:Autoimmune thyroid disease	2	16.67	0.02	CGA,CTLA4	5	19	3184	67.03	0.58	0.58	19.24
day0+day12 Hippocampus												
	GO:0002125~maternal aggressive behavior	2	9.09	0.01	AVP,OXT	18	3	8330	308.52	0.93	0.93	8.28
	GO:0002118~aggressive behavior	2	9.09	0.01	AVP,OXT	18	3	8330	308.52	0.93	0.93	8.28
	GO:0046903~secretion	4	18.18	0.01	CGA,LAT,AVP,OXT	18	207	8330	8.94	0.97	0.82	10.64
	GO:0045924~regulation of female receptivity	2	9.09	0.01	AVP,OXT	18	4	8330	231.39	0.97	0.69	10.88
	GO:0060180~female mating behavior	2	9.09	0.01	AVP,OXT	18	4	8330	231.39	0.97	0.69	10.88
	GO:0060746~parental behavior	2	9.09	0.01	AVP,OXT	18	5	8330	185.11	0.99	0.67	13.41
	GO:0042711~maternal behavior	2	9.09	0.01	AVP,OXT	18	5	8330	185.11	0.99	0.67	13.41
	GO:0042538~hyperosmotic salinity response	2	9.09	0.01	AVP,OXT	18	6	8330	154.26	0.99	0.65	15.87
	GO:0033057~reproductive behavior in a multicellular organism	2	9.09	0.01	AVP,OXT	18	6	8330	154.26	0.99	0.65	15.87
	GO:0007625~grooming behavior	2	9.09	0.01	AVP,OXT	18	7	8330	132.22	1.00	0.64	18.26
	GO:0050891~multicellular organismal water homeostasis	2	9.09	0.01	AVP,OXT	18	7	8330	132.22	1.00	0.64	18.26
	GO:0003078~regulation of natriuresis	2	9.09	0.01	AVP,OXT	18	7	8330	132.22	1.00	0.64	18.26
	GO:0044062~regulation of excretion	2	9.09	0.02	AVP,OXT	18	8	8330	115.69	1.00	0.63	20.58
	GO:0007617~mating behavior	2	9.09	0.02	AVP,OXT	18	8	8330	115.69	1.00	0.63	20.58

Category	Term	Count	%	PValue	Genes	List Total	Pop Hits	Pop Total	Fold Enrichment	Bonferroni	Benjamini	FDR
BP	GO:0051969~regulation of transmission of nerve impulse	3	13.64	0.02	LAMA2,AVP,OXT	18	100	8330	13.88	1.00	0.61	21.76
	GO:0007620~copulation	2	9.09	0.02	AVP,OXT	18	9	8330	102.84	1.00	0.58	22.84
	GO:0031644~regulation of neurological system process	3	13.64	0.02	LAMA2,AVP,OXT	18	105	8330	13.22	1.00	0.56	23.60
	GO:0035176~social behavior	2	9.09	0.02	AVP,OXT	18	10	8330	92.56	1.00	0.55	25.03
	GO:0019098~reproductive behavior	2	9.09	0.02	AVP,OXT	18	10	8330	92.56	1.00	0.55	25.03
	GO:0009651~response to salt stress	2	9.09	0.02	AVP,OXT	18	11	8330	84.14	1.00	0.55	27.16
	GO:0030104~water homeostasis	2	9.09	0.02	AVP,OXT	18	11	8330	84.14	1.00	0.55	27.16
	GO:0006972~hyperosmotic response	2	9.09	0.02	AVP,OXT	18	12	8330	77.13	1.00	0.56	29.23
	GO:0007618~mating	2	9.09	0.03	AVP,OXT	18	14	8330	66.11	1.00	0.58	33.20
	GO:0045777~positive regulation of blood pressure	2	9.09	0.03	AVP,OXT	18	16	8330	57.85	1.00	0.61	36.94
	GO:0051186~cofactor metabolic process	3	13.64	0.03	CIAO1,MOCOS,GPX3	18	146	8330	9.51	1.00	0.62	39.42
	GO:0051705~behavioral interaction between organisms	2	9.09	0.04	AVP,OXT	18	18	8330	51.42	1.00	0.61	40.48
	GO:0050806~positive regulation of synaptic transmission	2	9.09	0.04	LAMA2,OXT	18	19	8330	48.71	1.00	0.60	42.17
	GO:0051971~positive regulation of transmission of nerve impulse	2	9.09	0.04	LAMA2,OXT	18	20	8330	46.28	1.00	0.60	43.82
	GO:0031646~positive regulation of neurological system process	2	9.09	0.04	LAMA2,OXT	18	22	8330	42.07	1.00	0.62	46.97
	GO:0051240~positive regulation of multicellular organismal process	3	13.64	0.05	LAMA2,AVP,OXT	18	169	8330	8.21	1.00	0.62	48.19
	GO:0006970~response to osmotic stress	2	9.09	0.05	AVP,OXT	18	27	8330	34.28	1.00	0.66	54.10
	GO:0032846~positive regulation of homeostatic process	2	9.09	0.07	AVP,OXT	18	33	8330	28.05	1.00	0.72	61.40
	GO:0044057~regulation of system process	3	13.64	0.07	LAMA2,AVP,OXT	18	210	8330	6.61	1.00	0.71	62.44
	GO:0043279~response to alkaloid	2	9.09	0.07	AVP,OXT	18	38	8330	24.36	1.00	0.74	66.60
MF	GO:0005179~hormone activity	3	13.64	0.00	CGA,AVP,OXT	14	51	7910	33.24	0.14	0.14	2.90
	GO:0031895~V1B vasopressin receptor binding	2	9.09	0.00	AVP,OXT	14	2	7910	565.00	0.15	0.08	3.13
	GO:0031855~oxytocin receptor binding	2	9.09	0.00	AVP,OXT	14	2	7910	565.00	0.15	0.08	3.13
	GO:0031893~vasopressin receptor binding	2	9.09	0.00	AVP,OXT	14	3	7910	376.67	0.22	0.08	4.66
	GO:0031894~V1A vasopressin receptor binding	2	9.09	0.00	AVP,OXT	14	3	7910	376.67	0.22	0.08	4.66
	GO:0005184~neuropeptide hormone activity	2	9.09	0.02	AVP,OXT	14	10	7910	113.00	0.57	0.19	14.72
CC	GO:0005625~soluble fraction	3	13.64	0.08	CGA,AVP,GPX3	17	230	7836	6.01	1.00	1.00	57.24
KP	NIL											
day0+day12 Dorsal Hypothalamus												
	GO:0032413~negative regulation of ion transmembrane transporter activity	2	5.13	0.01	DRD2,PKD2	31	4	8330	134.35	1.00	1.00	19.09
	GO:0045022~early endosome to late endosome transport	2	5.13	0.03	FAM160A2,LYST	31	8	8330	67.18	1.00	1.00	34.54
	GO:0032410~negative regulation of transporter activity	2	5.13	0.03	DRD2,PKD2	31	9	8330	59.71	1.00	1.00	37.92

Category	Term	Count	%	PValue	Genes	List Total	Pop Hits	Pop Total	Fold Enrichment	Bonferroni	Benjamini	FDR
BP	GO:0051282~regulation of sequestering of calcium ion	2	5.13	0.04	DRD2,PKD2	31	10	8330	53.74	1.00	0.99	41.12
	GO:0051283~negative regulation of sequestering of calcium ion	2	5.13	0.04	DRD2,PKD2	31	10	8330	53.74	1.00	0.99	41.12
	GO:0051209~release of sequestered calcium ion into cytosol	2	5.13	0.04	DRD2,PKD2	31	10	8330	53.74	1.00	0.99	41.12
	GO:0060402~calcium ion transport into cytosol	2	5.13	0.05	DRD2,PKD2	31	14	8330	38.39	1.00	1.00	52.37
	GO:0032412~regulation of ion transmembrane transporter activity	2	5.13	0.05	DRD2,PKD2	31	15	8330	35.83	1.00	0.99	54.83
	GO:0060401~cytosolic calcium ion transport	2	5.13	0.05	DRD2,PKD2	31	15	8330	35.83	1.00	0.99	54.83
	GO:0034765~regulation of ion transmembrane transport	2	5.13	0.06	DRD2,PKD2	31	16	8330	33.59	1.00	0.99	57.16
	GO:0022898~regulation of transmembrane transporter activity	2	5.13	0.06	DRD2,PKD2	31	17	8330	31.61	1.00	0.99	59.38
	GO:0034762~regulation of transmembrane transport	2	5.13	0.06	DRD2,PKD2	31	18	8330	29.86	1.00	0.98	61.47
	GO:0007243~protein kinase cascade	4	10.26	0.08	MAPK13,DRD2,MKNK2,PKD2	31	281	8330	3.83	1.00	0.99	70.10
	GO:0032409~regulation of transporter activity	2	5.13	0.08	DRD2,PKD2	31	23	8330	23.37	1.00	0.99	70.45
MF	NIL											
CC	NIL											
KP	NIL											
	day0+day12 Ventral Hypothalamus											
BP	GO:0048015~phosphoinositide-mediated signaling	4	5.88	0.01	GNA15,PIK3C2G,PIK3C2B,HTR2A	53	60	8330	10.48	0.97	0.97	8.55
	GO:0030199~collagen fibril organization	3	4.41	0.01	LUM,COL1A1,COL11A2	53	20	8330	23.58	0.98	0.84	9.42
	GO:0019932~second-messenger-mediated signaling	5	7.35	0.01	LAT,GNA15,PIK3C2G,PIK3C2B,HTR2A	53	139	8330	5.65	1.00	0.86	14.67
	GO:0060249~anatomical structure homeostasis	4	5.88	0.01	TNKS1BP1,ANKRD11,TEP1,COL11A2	53	77	8330	8.16	1.00	0.81	16.30
	GO:0009628~response to abiotic stimulus	6	8.82	0.03	XRCC4,NPHP4,CUL5,THBD,USP1,COL1A1	53	282	8330	3.34	1.00	0.97	36.36
	GO:0042592~homeostatic process	8	11.76	0.04	TNKS1BP1,GNA15,CUL5,ANKRD11,TEP1,CCDC47,COL11A2,HTR2A	53	519	8330	2.42	1.00	0.98	45.73
	GO:0048871~multicellular organismal homeostasis	3	4.41	0.05	ANKRD11,COL11A2,HTR2A	53	58	8330	8.13	1.00	0.98	53.11
	GO:0006817~phosphate transport	2	2.94	0.06	SLC17A7,MARCO	53	10	8330	31.43	1.00	0.99	59.89
	GO:0060323~head morphogenesis	2	2.94	0.06	ANKRD11,COL1A1	53	10	8330	31.43	1.00	0.99	59.89
	GO:0046854~phosphoinositide phosphorylation	2	2.94	0.07	PIK3C2G,PIK3C2B	53	11	8330	28.58	1.00	0.98	63.40
	GO:0009314~response to radiation	4	5.88	0.07	XRCC4,NPHP4,THBD,USP1	53	153	8330	4.11	1.00	0.98	65.26
	GO:0010171~body morphogenesis	2	2.94	0.07	ANKRD11,COL1A1	53	12	8330	26.19	1.00	0.98	66.60
	GO:0046834~lipid phosphorylation	2	2.94	0.07	PIK3C2G,PIK3C2B	53	12	8330	26.19	1.00	0.98	66.60
	GO:0060322~head development	2	2.94	0.07	ANKRD11,COL1A1	53	12	8330	26.19	1.00	0.98	66.60
	GO:0010165~response to X-ray	2	2.94	0.08	XRCC4,THBD	53	13	8330	24.18	1.00	0.98	69.52

Category	Term	Count	%	PValue	Genes	List Total	Pop Hits	Pop Total	Fold Enrichment	Bonferroni	Benjamini	FDR
	GO:0048705~skeletal system morphogenesis	3	4.41	0.08	ANKRD11,COL1A1,COL11A2	53	76	8330	6.20	1.00	0.97	70.98
	GO:0006259~DNA metabolic process	6	8.82	0.08	POLL,TNKS1BP1,XRCC4,FANCM,USP1,TEP1	53	374	8330	2.52	1.00	0.97	71.56
	GO:0030198~extracellular matrix organization	3	4.41	0.09	LUM,COL1A1,COL11A2	53	83	8330	5.68	1.00	0.97	76.49
	GO:0016445~somatic diversification of immunoglobulins	2	2.94	0.10	POLL,XRCC4	53	16	8330	19.65	1.00	0.97	76.83
	GO:0031532~actin cytoskeleton reorganization	2	2.94	0.10	RICTOR,LPIN1	53	16	8330	19.65	1.00	0.97	76.83
MF	GO:0035005~phosphatidylinositol-4-phosphate 3-kinase activity	2	2.94	0.02	PIK3C2G,PIK3C2B	53	3	7910	99.50	0.96	0.96	21.39
	GO:0005201~extracellular matrix structural constituent	3	4.41	0.05	LUM,COL1A1,COL11A2	53	54	7910	8.29	1.00	0.98	45.60
	GO:0016307~phosphatidylinositol phosphate kinase activity	2	2.94	0.06	PIK3C2G,PIK3C2B	53	9	7910	33.17	1.00	0.96	51.44
	GO:0035004~phosphoinositide 3-kinase activity	2	2.94	0.06	PIK3C2G,PIK3C2B	53	10	7910	29.85	1.00	0.94	55.19
	GO:0016303~1-phosphatidylinositol-3-kinase activity	2	2.94	0.06	PIK3C2G,PIK3C2B	53	10	7910	29.85	1.00	0.94	55.19
CC	GO:0005583~fibrillar collagen	3	4.41	0.00	LUM,COL1A1,COL11A2	45	10	7836	52.24	0.16	0.16	1.55
	GO:0005581~collagen	3	4.41	0.01	LUM,COL1A1,COL11A2	45	30	7836	17.41	0.79	0.54	13.18
	GO:0005886~plasma membrane	19	27.94	0.05	GNA15,NKD1,BST2,PHKB,PIK3C2B,CACNG6,LIFR,SLC17A7,MARCO,LAT,TNFRSF1B,THBD,RAB19,EFR3A,PAK1,COL1A1,UNC13C,TES,HTR2A	45	2220	7836	1.49	1.00	0.88	44.12
	GO:0005942~phosphoinositide 3-kinase complex	2	2.94	0.05	PIK3C2G,PIK3C2B	45	10	7836	34.83	1.00	0.83	47.92
	GO:0044459~plasma membrane part	14	20.59	0.06	GNA15,BST2,PHKB,CACNG6,LIFR,SLC17A7,MARCO,LAT,THBD,RAB19,PAK1,UNC13C,HTR2A,TES	45	1491	7836	1.64	1.00	0.80	51.94
	GO:0044420~extracellular matrix part	3	4.41	0.09	LUM,COL1A1,COL11A2	45	92	7836	5.68	1.00	0.87	68.10
KP	hsa03450:Non-homologous end-joining	2	2.94	0.07	POLL,XRCC4	23	10	3184	27.69	0.98	0.98	49.62
	hsa04666:Fc gamma R-mediated phagocytosis	3	4.41	0.08	LAT,PAK1,AKT3	23	68	3184	6.11	0.99	0.90	55.60
	hsa04510:Focal adhesion	4	5.88	0.10	COL1A1,PAK1,COL11A2,AKT3	23	161	3184	3.44	1.00	0.85	63.35
day0+day12 Anterior Pituitary												
BP	NIL											
MF	GO:0008081~phosphoric diester hydrolase activity	2	20.00	0.06	PLD3,ENPP2	8	66	7910	29.96	0.89	0.89	41.15
	GO:0030247~polysaccharide binding	2	20.00	0.09	SERPINE2,ENPP2	8	110	7910	17.98	0.98	0.84	58.78
	GO:0001871~pattern binding	2	20.00	0.09	SERPINE2,ENPP2	8	110	7910	17.98	0.98	0.84	58.78
CC	GO:0005576~extracellular region	4	40.00	0.09	TMEFF2,SERPINE2,CRB1,ENPP2	9	1081	7836	3.22	0.95	0.95	54.58
KP	NIL											

Category	Term	Count	%	PValue	Genes	List Total	Pop Hits	Pop Total	Fold Enrichment	Bonferroni	Benjamini	FDR

* All terms with p<0.10 are included

BP	Gene Ontology Biological Process
MF	Gene Ontology Molecular function
CC	Gene Ontology Cellular component
KP	KEGG Pathway
