# Supplementary material for: Gene expression patterns in four brain areas associate with quantitative measure of estrous behavior in dairy cows
Source: BMC Genomics. 2011 Apr 19;12:200. doi: 10.1186/1471-2164-12-200 (PMC3110153; doi:10.1186/1471-2164-12-200)
Supplement: Additional file 4 — Collection of brain samples and pituitary from the experimental cows. Procedure followed for collection of anterior pituitary and brain samples: amygdala, hippocampus, dorsal hypothalamus and ventral hypothalamus (with pictures). [file 1471-2164-12-200-S4.PDF]

Collection of brain samples and pituitary from the experimental cows

The cow is gently guided (stress free) to the place where it will be euthanized (the calving stable), and held in place between 2 barriers. Blood is collected from the udder vein into 3 EDTA containing tubes (10ml) and 1 heparin containing tube (10ml) and put on ice. Two of the EDTA tubes are directly stored at -20 ˚C and the other 2 (1 EDTA and 1 heparin tube) are centrifuged (10 min 2000 rpm) and the plasma is stored at -80 ˚C.

The cow is euthanized by injecting 20 ml of T61 into the udder vein. After the cow has fallen and the pupils stop reacting, the jugular vein is cut and the cow is bled. The head is removed with 1 ear (with tag number) remaining attached to the body and the other to the head. The roof of the skull is cut off with an axe to free the brain for removal (Figure 1). Samples for RNA-isolation are taken from the left-side of the cow brain. The right half is fixed in formalin.

Figure 1. Removal of the brain

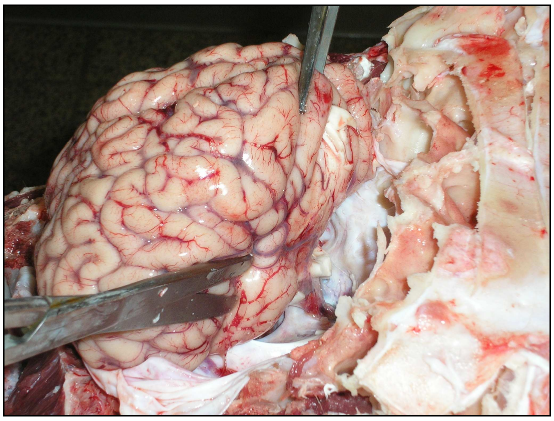

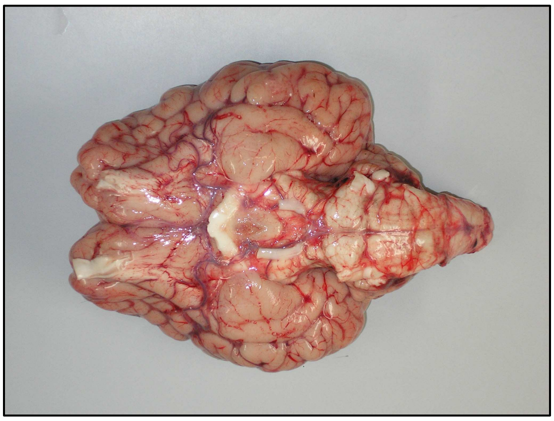

Collection of Pituitary samples

Detach the pituitary from the rest of the brain and cut in half (left and right sides). In Figure 2, label 9a represents the Anterior pituitary (Adenohypophysis) and 9b represents the Posterior pituitary (Neurohypophysis).

Figure 2. Collection of Pituitary gland

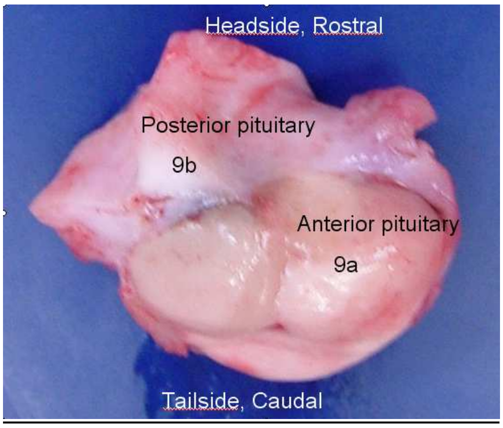

Collection of brain samples

Place the brain in a special box to keep its position fixed. Place 3 knives (9-10 mm apart) at designated slots on the box: the middle knife is placed caudal to the optic chiasm against the two endings of the arches immediately before the pituitary stalk (Figure 3). Slice through the brain and place the slices on the table: the rostral slice with its front side on the table (slice 1), and the caudal slice with its back side on the table (slice 2).

Figure 3. Taking slices of the brain

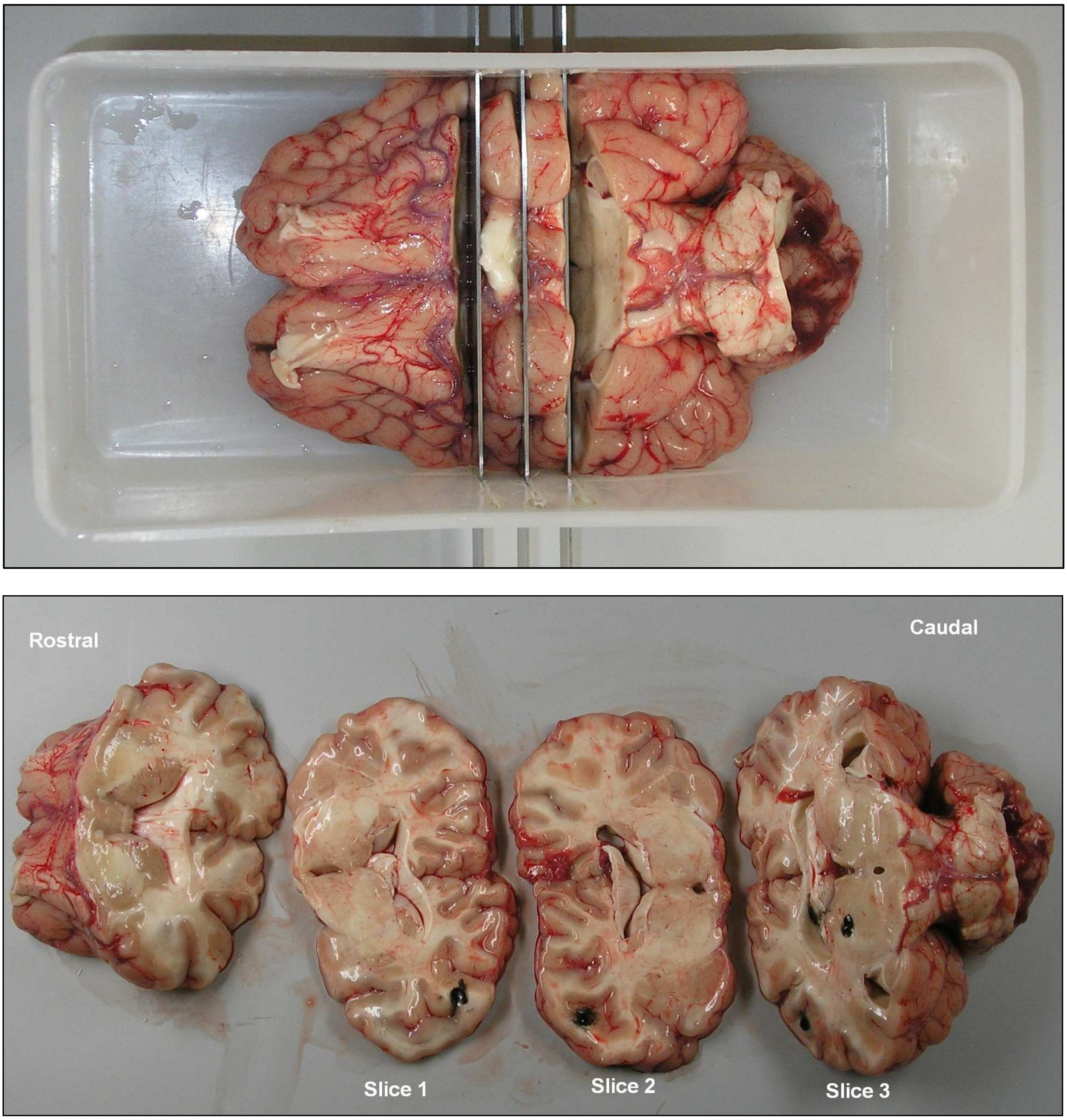

Brain areas collected from Slice 1 (front side on the table, RNA from left part)

Hypothalamus:
A 0.5 cm piece is cut right above the optic chiasm to the bottom of the lateral ventricles. In figure 4, label 1a represents posterior (dorsal) hypothalamus and 1b represents inferior (ventral) hypothalamus.

Figure 4. Brain areas collected from slice 1

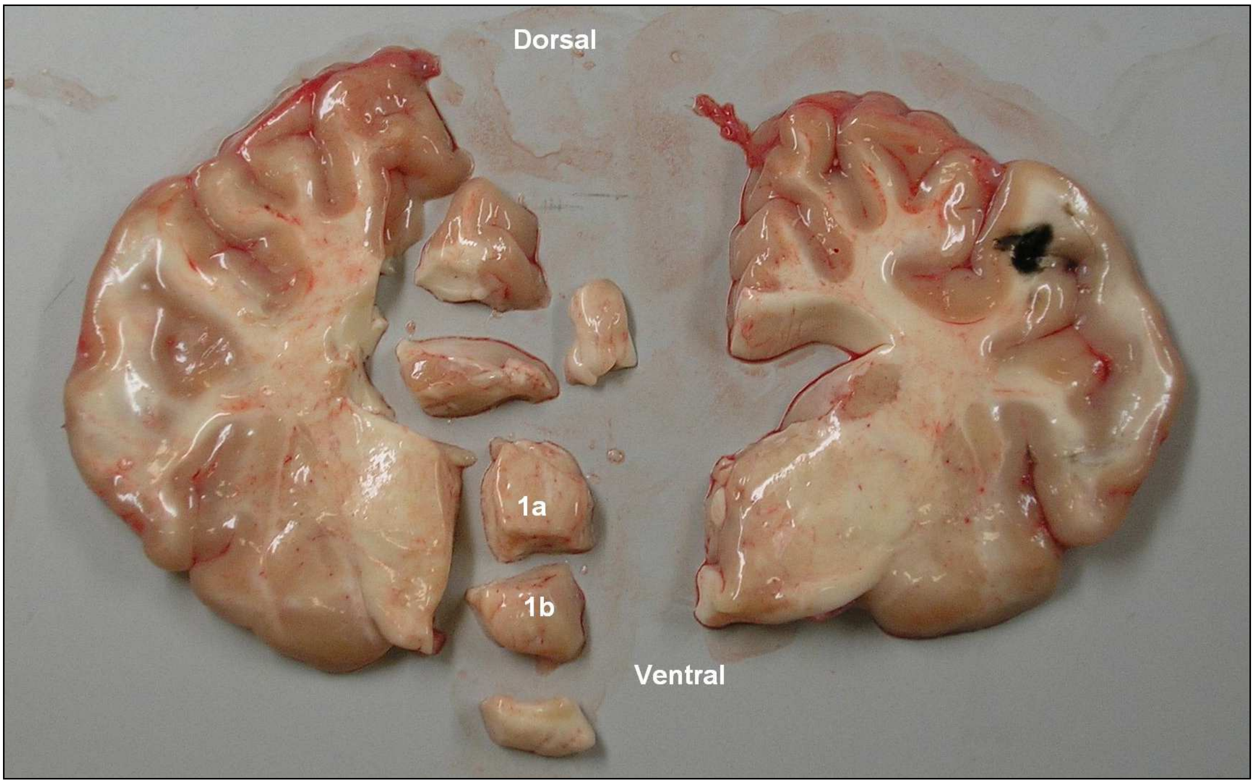

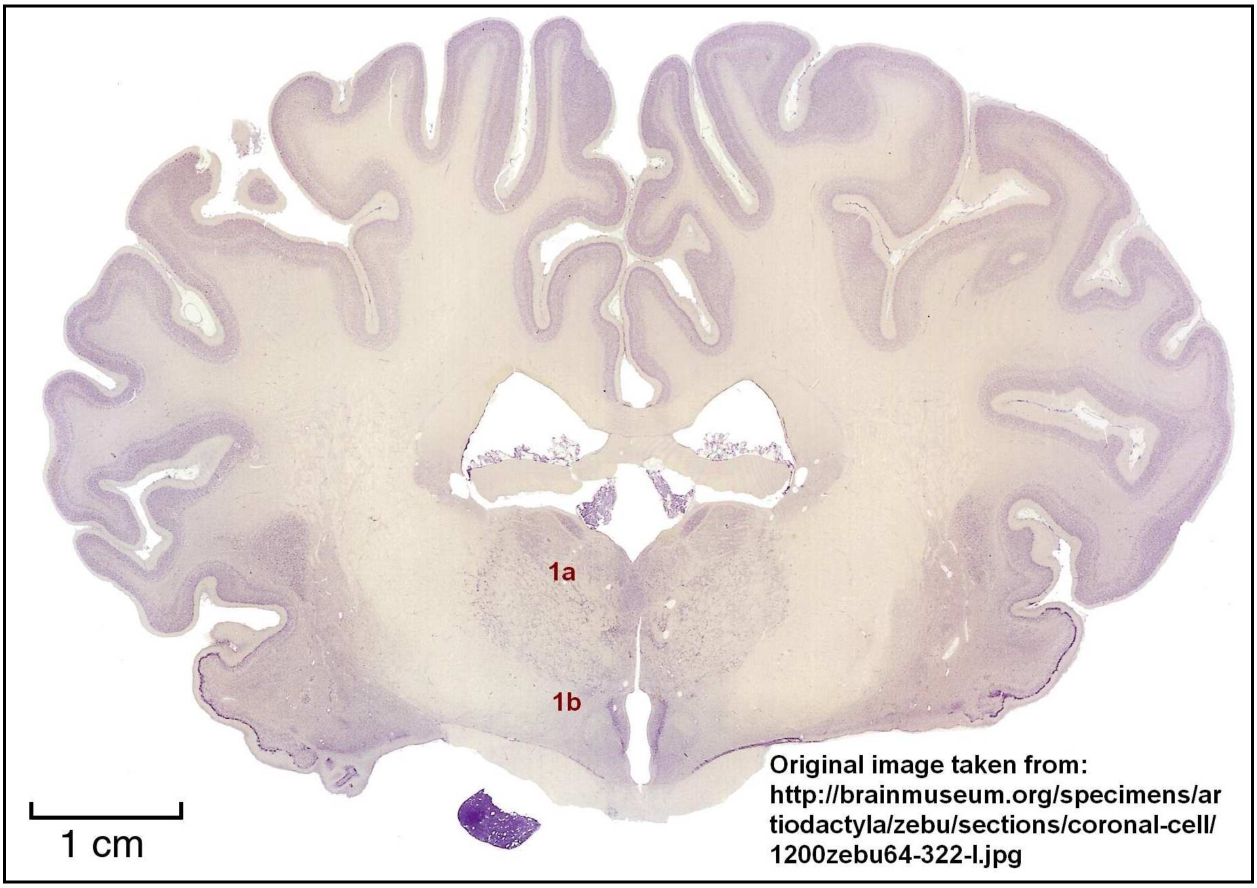

Brain areas collected from Slice 2 (back side on the table, RNA from right part)

Hypothalamus:
Similar to slice 1, a 0.5 cm piece is cut right above the optic chiasm to the bottom of the lateral ventricles. In figure 5, label 4a represents posterior (dorsal) hypothalamus and 4b represents inferior (ventral) hypothalamus.

Amygdala:
Isolate lobus piriformus (complete lateral-ventral lobe). The slice is inverted compared to slice 1, so the area for RNA isolation seems to be from the right half of the brain but is actually from the left. In figure 5, label 5a represents posterior / dorsal amygdala and 5b represents inferior / ventral (basal) amygdala.

Figure 5. Brain areas collected from slice 2

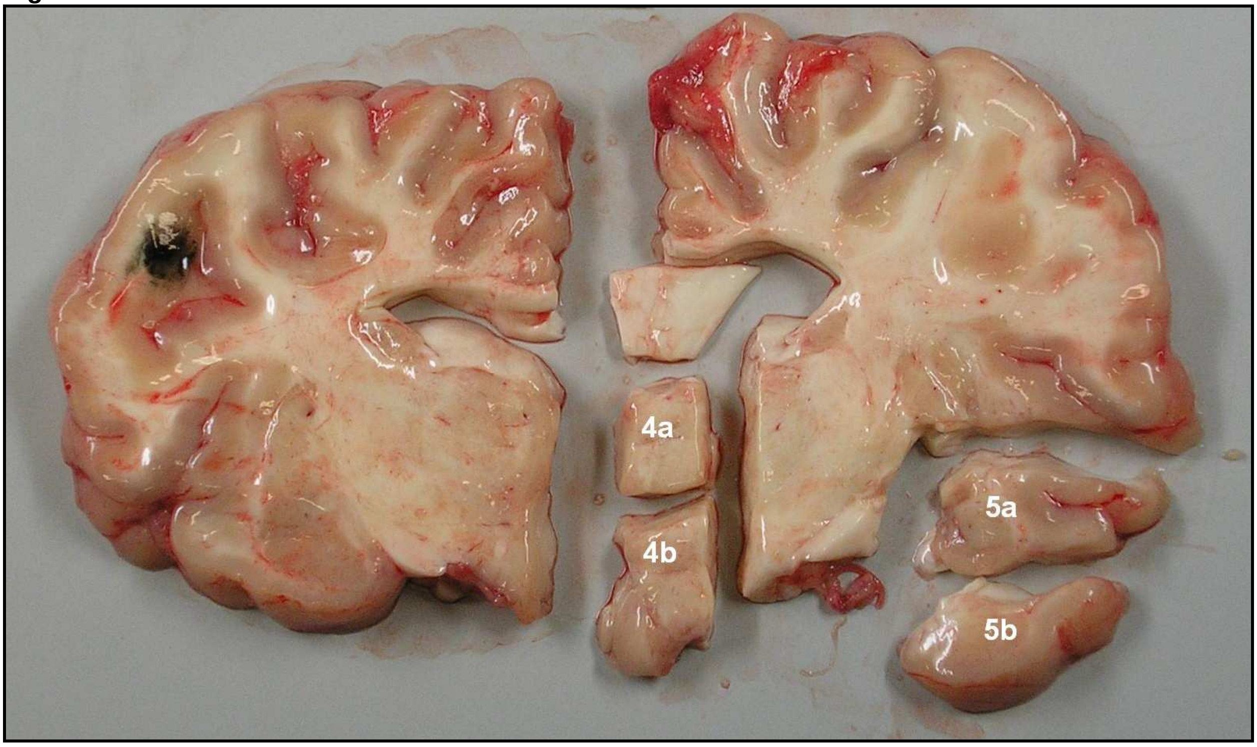

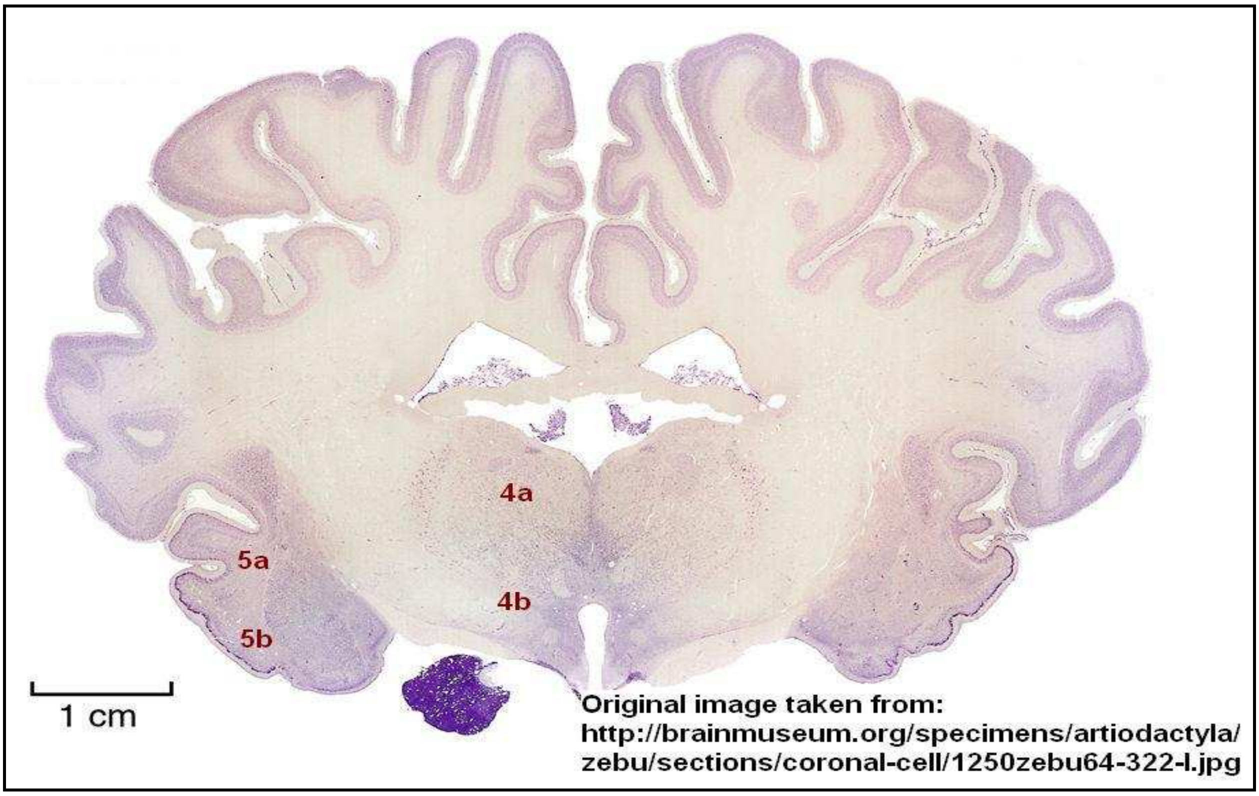

Brain areas collected from Slice 3 (back side on the table, RNA from right part)

Hippocampus:
From the caudal remnant (slice 3), the Ammon's horn is isolated and cut in half. In figure 6, label 7a represents posterior (dorsal) part of the hippocampus and 7b represents inferior (ventral) part of the hippocampus with the dentate gyrus

Figure 6. Brain areas collected from slice 3

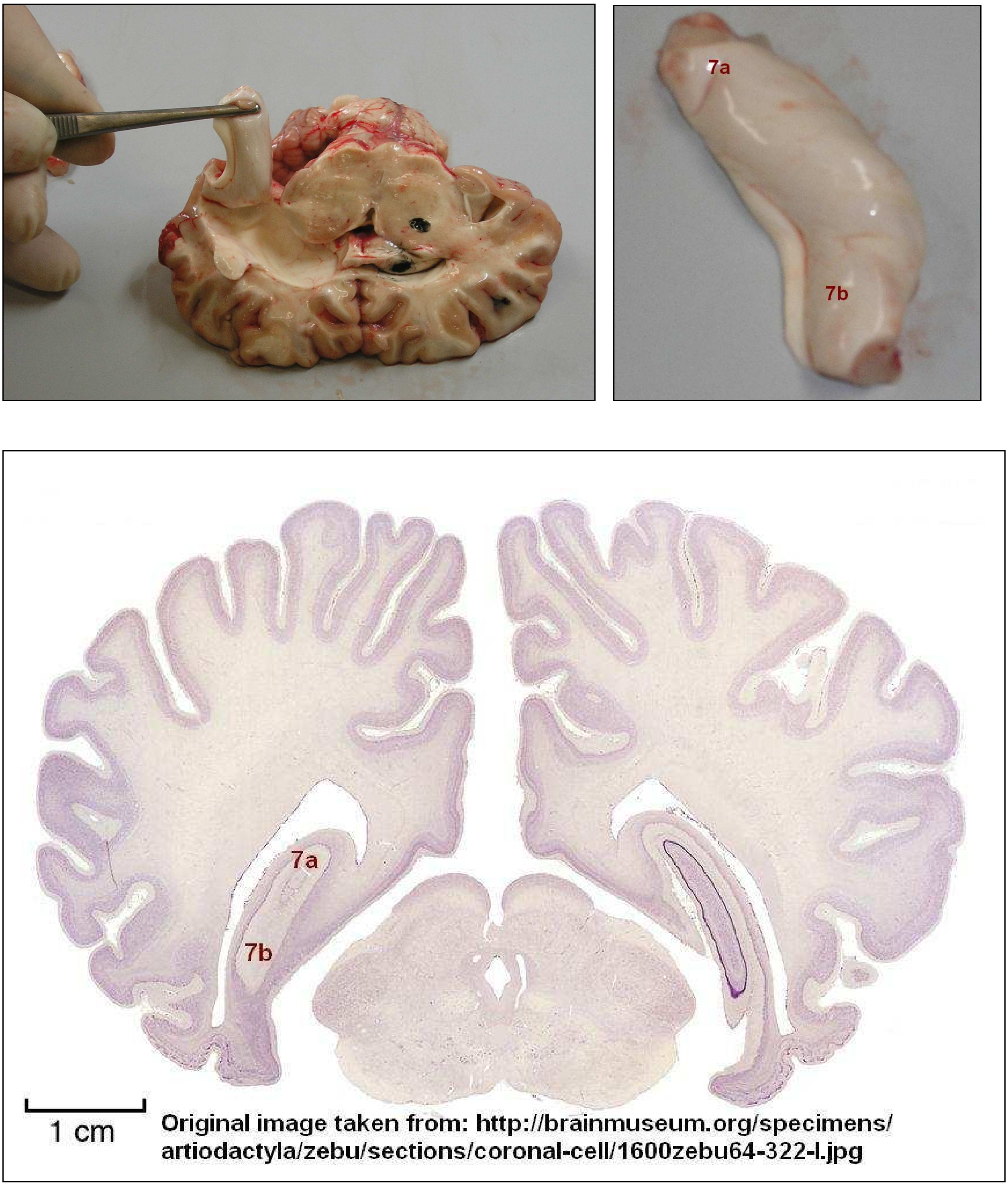

* The 3 images of the histological sections of the brain used in this document have been obtained from the website http://brainmuseum.org. These images are from the University of Wisconsin and Michigan State Comparative Mammalian Brain Collections, as well as from those at the National Museum of Health and Medicine. We also refer to the fact that preparation of these images and specimens have been funded by the National Science Foundation, as well as by the National Institutes of Health.
